# Unlocking precision in aptamer engineering: a case study of the thrombin binding aptamer illustrates why modification size, quantity, and position matter

**DOI:** 10.1093/nar/gkae729

**Published:** 2024-09-01

**Authors:** Makay T Murray, Stacey D Wetmore

**Affiliations:** Department of Chemistry and Biochemistry, University of Lethbridge, 4401 University Drive West, Lethbridge, Alberta T1K 3M4, Canada; Department of Chemistry and Biochemistry, University of Lethbridge, 4401 University Drive West, Lethbridge, Alberta T1K 3M4, Canada

## Abstract

The thrombin binding aptamer (TBA) is a prototypical platform used to understand the impact of chemically-modified nucleotides on aptamer stability and target affinity. To provide structural insight into the experimentally-observed effects of modification size, location, and number on aptamer performance, long time-scale molecular dynamics (MD) simulations were performed on multiple binding orientations of TBA–thrombin complexes that contain a large, flexible tryptophan thymine derivative (T-W) or a truncated analogue (T-K). Depending on modification position, T-W alters aptamer–target binding orientations, fine-tunes aptamer–target interactions, strengthens networks of nucleic acid–protein contacts, and/or induces target conformational changes to enhance binding. The proximity and 5′-to-3′ directionality of nucleic acid structural motifs also play integral roles in the behavior of the modifications. Modification size can differentially influence target binding by promoting more than one aptamer–target binding pose. Multiple modifications can synergistically strengthen aptamer–target binding by generating novel nucleic acid–protein structural motifs that are unobtainable for single modifications. By studying a diverse set of modified aptamers, our work uncovers design principles that must be considered in the future development of aptamers containing chemically-modified nucleotides for applications in medicine and biotechnology, highlighting the value of computational studies in nucleic acids research.

## Graphical Abstract

**Graphical Abstract:**
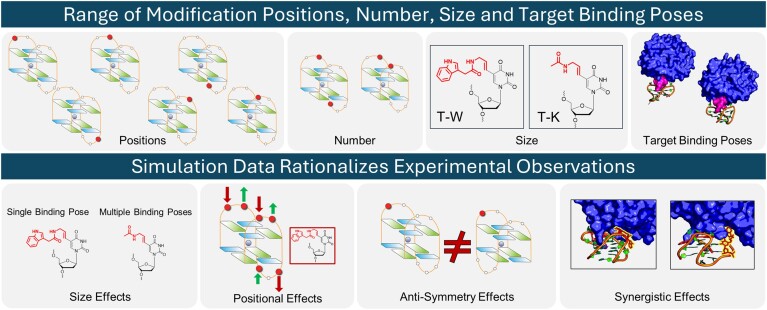

## Introduction

Aptamers are single-stranded nucleic acids, typically 15–100 nucleotides long, that are designed to bind with high affinity and specificity to a wide range of targets (e.g. small molecules, proteins, metals, or cells) (1). As a result, aptamers have been used in a breadth of applications such as allosteric protein inhibitors (2), conformational switches (3), metal detectors (4,5), antivenoms (6) and food safety (7). Aptamers have also been used as therapeutics (8–11), rivaling antibodies due to their remarkable target affinity and specificity, ease and low cost of manufacturing, and enhanced chemical stability *in vivo*.

Although aptamers can be solely composed of canonical nucleotides, aptamer function can be further tuned using chemical modifications, which have the capability to increase stability and enhance on-target binding (12–21). Modifications have been introduced to all components of nucleotides, including the phosphate or deoxyribose in the nucleic acid backbone (14–18), as well as the nucleobases (14–16,19–22). In some applications, an entire nucleotide unit has been altered (23–26). As evidence of the power of modifications, aptamers containing modified nucleotides are being used as drugs to treat age-related macular degeneration (Pegaptanib (Macugen) (27) and Avacincaptad pegol (Izervay) (28) and as biosensors to detect breast cancer (HeA2) (29).

**Figure 1.**
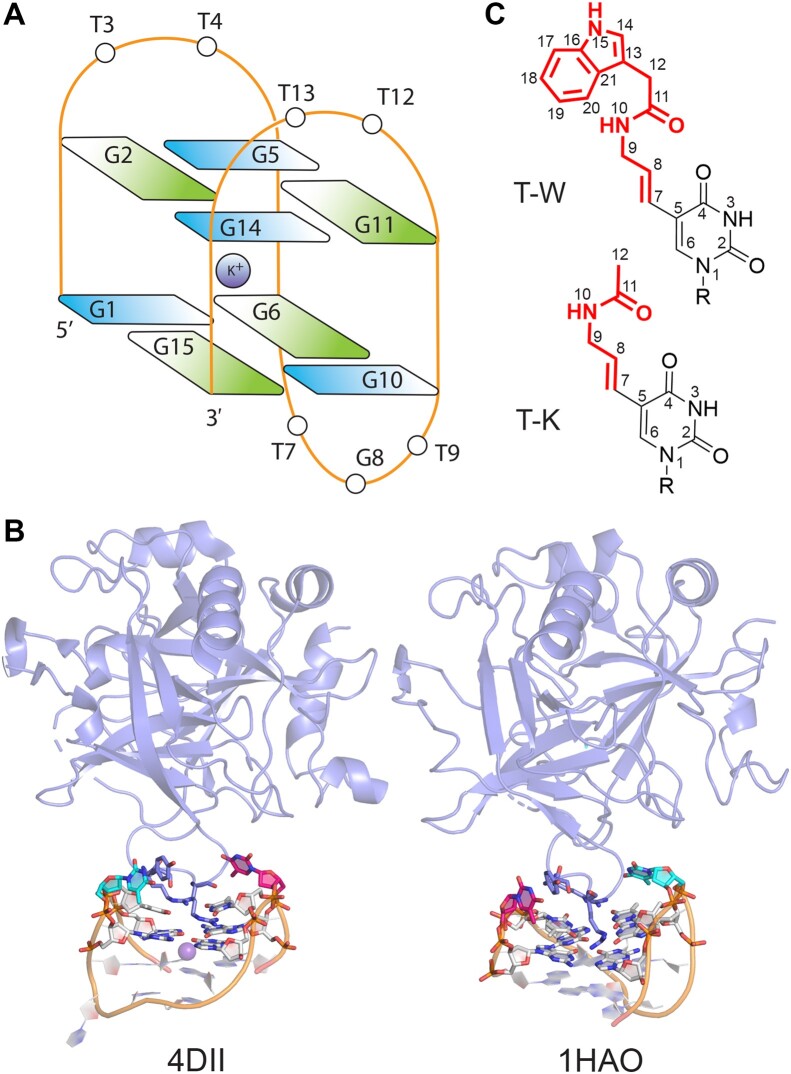
(**A**) Schematic of TBA, with G coloured according to glycosidic bond orientation (*syn* in blue and *anti* in green). (**B**) X-ray crystal structures of canonical TBA bound to thrombin (PDB IDs: 4DII (left) and 1HAO (right)). (**C**) Chemical structure and numbering of the T-W (5-(indolyl-3-acetyl-3-amino-1-propenyl)-2′-deoxyuridine) and T-K (5-(methyl-3-acetyl-3-amino-1-propenyl)-2′-deoxyuridine) modifications considered in the present study.

Despite the promise of chemical modifications, their impact on aptamer structure and function is currently poorly understood, which prohibits the rational development of highly efficient aptamers for diverse applications. In attempts to address this void in information, the thrombin binding aptamer (TBA, 5′–GGTTGG–TGT–GGTTGG–3′) has been widely investigated as a model system to understand the effects of modifications (12–15,17–18,20,22–26,30–82). TBA targets a protein (thrombin) involved in the blood clotting cascade (83) and is composed of a guanine quadruplex (GQ) containing two guanine tetrads, two TT loops, and a TGT loop (Figure 1A) (76,84). Canonical TBA is well accepted to bind to exosite I of thrombin via the two TT loops (Figure 1B). Many different modifications have been introduced into various nucleotide positions in TBA to investigate the effects of chemical alterations on the *in vivo* half-life, target binding affinity, and/or prothrombin time (12–15,17,18,20,22–26,30–82,85). These studies collectively illustrate that modification type and location have diverse impacts on aptamer stability and function. However, since few crystal structures of modified TBA are available (31,32,36,71,73,78), atomic-level rationalizations of experimental observations necessary for improved aptamer design are currently lacking.

Computational techniques can readily provide dynamical atomic-level information about the structure and target binding of aptamers. Indeed, molecular dynamics (MD) simulations on (unmodified) isolated and thrombin-bound TBA have been used to provide insight into aptamer folding (80,86,87) and cation uptake (74,79,88), as well as the conformational dynamics of the aptamer–protein complex (89). However, relative to the wealth of experimental work, fewer computational studies have considered modified TBA (22,35,39,42,50–52,69,74,80,81,86,88–90). Although the computational approach adopted in studies of modified TBA is highly varied, a single, short (1–100 ns) MD simulation has often been used despite microsecond simulation times and replicas being essential for an accurate description of nucleic acid structure (91–93). Additionally, to the best of our knowledge, no computational study has systematically investigated the impact of large, flexible modifications or thoroughly explored the synergistic effects of multiple chemical alterations to TBA. Thus, accurate explanations for the impact of diverse modifications on TBA structure and function are still required.

To fill gaps in our knowledge regarding how to exploit modifications in aptamer design, the present work considers the case study of two thymine modifications that were previously synthesized to capitalize on known favorable protein–protein interactions to enhance aptamer–target binding (Figure 1C), namely T-W (5-(indolyl-3-acetyl-3-amino-1-propenyl)-2′-deoxyuridine) and T-K (5-(methyl-3-acetyl-3-amino-1-propenyl)-2′-deoxyuridine) (31). These modifications were specifically chosen due to their large size and flexibility coupled with the broader importance of C5 modifications to T for aptamer design (20,22,31,38,44,45,66,85), particularly for TBA as the TT loops are directly involved in target binding (Figure 1B). Furthermore, the T-W and T-K modifications are a good case study since an abundance of accurate experimental data is available, including high-resolution crystal structures of T4 modified TBAs as well as melting temperatures and thrombin binding affinities for a range of modification positions and numbers (31). The experimental data highlights that the introduction of the T-W modifications at T4, T7 or T12 enhances canonical TBA–thrombin binding, while T-W at T3, T9 or T13 decreases canonical TBA binding affinity (Table 1). Furthermore, truncation of T-W at T4 to T-K or the simultaneous incorporation of two T-W modifications (T4 and T7 or T12) substantially increases TBA affinity for thrombin (Table 1). To rationalize this differential impact of individual and pairs of modifications at T sites of TBA on thrombin binding, the present work uses long timescale MD simulations. Our work reveals fundamental structural information about the effects of modification type and position as well as the synergistic interplay of multiple modifications on TBA function. In addition to filling a void in the current scarcity of atomic-level information about chemically-modified aptamer structure and function, the insights obtained are critical for the rational design of new and improved aptamers for a host of applications in medicine (1,2,6,8–10,12) and biotechnology (1,3,7,12).

**Table 1.** Experimental absolute and relative binding affinities for canonical and modified TBA, as well as thrombin binding orientations considered in the present work

			Binding orientation^c^	
Aptamer	*K*_D_ (M)^a^	Relative *K*_D_^a,b^	4DII	1HAO
**TBA**	2.8 × 10^–9^	1.0	X	X
**T3W**	3.9 × 10^–9^	0.7	X	
**T4W**	1.0 × 10^–9^	2.8	X	X
**T7W**	1.7 × 10^–9^	1.7	X	X
**T9W**	5.1 × 10^–9^	0.5	X	
**T12W**	1.3 × 10^–9^	2.2	X	X
**T13W**	5.4 × 10^–9^	0.5	X	
**T4WT7W**	3.2 × 10^–10^	8.7	X	X
**T4WT12W**	3.1 × 10^–10^	9.3	X	X
**T4K**	3.9 × 10^–10^	7.0	X	X

^a^Reported in (31).

^b^Relative *K*_D_ = *K*_D_^TBA^/*K*_D_^Modified TBA^.

^c^Binding orientation corresponds to the relative TBA–thrombin orientation in the crystal structure (PDB ID: 4DII or 1HAO) used to initiate MD simulations the present work (Figure 1B).

## Materials and methods

MD simulations were performed on canonical and modified TBA–thrombin complexes built from X-ray crystal structures of the canonical aptamer bound to the protein in two poses differing by a ∼180° rotation about the principal axis of TBA prior to binding (PDB ID: 4DII (2.05 Å) (76) and 1HAO (2.80 Å) (84), Figure 1B). Crystallographic water and molecules used for crystallization were removed, while unresolved protein residues (Supplementary Table S1) and the unresolved potassium ion central to the GQ in 1HAO was added. To generate the modified TBA–thrombin complexes considered in the present work (Supplementary Table S2), T4 of canonical TBA was replaced with the minimum energy conformation of T-K, while each T residue of TBA was successively replaced with the most stable T-W conformation (Supplementary Figure S1). In addition, select doubly-modified aptamers were investigated and the canonical TBA–thrombin complex was considered as a control. In our nomenclature, the modification at T4 is denoted T4-W (or T4-K), a TBA molecule with T4 replaced with T-W is denoted T4W, and the corresponding modified TBA–thrombin complex is denoted T4W_PDB ID_ (e.g. T4W_4DII_ is the T4W–thrombin complex with the binding orientation in PDB ID: 4DII). Thrombin residue numbering was used as per PDB ID: 4DII (76).

Each system was solvated in a truncated-octahedral TIP4P-EW water box such that the solute is at least 10 Å from the box face in any direction, neutralized with Na^+^, and NaCl added to reach 150 mM (see Supplementary Table S3 for water and ion counts). We note that the single K^+^ ion and NaCl concentration used mimics conditions of intercellular space where coagulation occurs, as well as crystallographic conditions and computational studies on other modified TBA–thrombin complexes, and thereby permits accurate comparisons to previous literature. The Amber force field (94) was used such that amino acids were modeled with ff14SB and nucleotides with OL15, while the parameters for ions were adopted from Joung and Cheatam (95). AMBER parameters for T-W and T-K were supplemented by the general amber forcefield (GAFF) as determined using antechamber, while charges were determined using the RESP fitting approach (Supplementary Figure S2). Each complex was sequentially minimized, heated to 310 K, and equilibrated. Finally, 1 μs MD simulations were performed in triplicate using the Amber18 software suite (96), while applying the periodic boundary conditions, the SHAKE algorithm, and using an NPT ensemble (310 K and 1 bar). As justified based on the root-mean-square deviation across the simulations (Supplementary Figure S3), frames were saved every 20 ps for analysis (60,000 frames total per system over all replicas). Representative structures were obtained by clustering with respect to the TBA–thrombin interface. Structural features were analyzed using CPPTRAJ and in-house scripts, and evaluated over all MD replicas. Occupancies for stacking interactions were evaluated based on the centers of mass of the two π systems being ≤ 5 Å and the coplanar angle falling within 0–30° or 150–180°, while hydrogen-bonding occupancies were evaluated using cutoffs for heavy-atom–to–heavy-atom distance ≤ 3.4 Å and heavy-atom–hydrogen–heavy-atom angle ≥ 130°. Statistical tests were performed to verify the significance of observed differences in these parameters (Supplementary Tables S4–S6).

Full details of the simulation and analysis protocols are provided in the Supplementary material.

## Results

### MD Simulations Initiated from Crystal Structures of the Canonical TBA–Thrombin Complex Rationalize the Experimentally-Observed Enhanced Binding of T4W and Demonstrate a Preference for a Single Binding Mode

**Figure 2.**
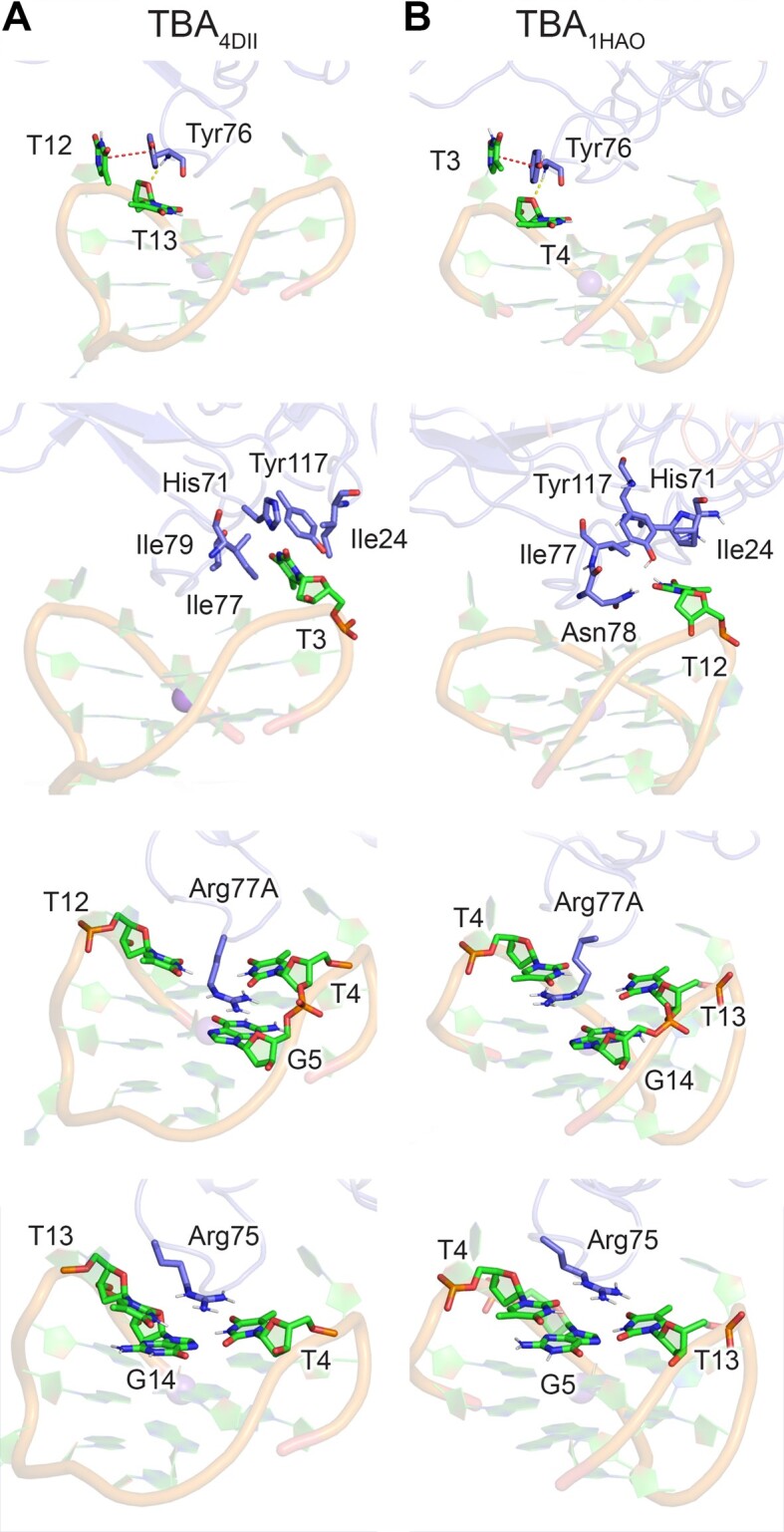
Key interactions across MD simulations on complexes between canonical TBA and thrombin initiated from PDB ID: (**A**) 4DII and (**B**) 1HAO. π–π interaction and hydrogen-bonding occupancies provided in Supplementary Figures S4, S5 and S8.

**Figure 3.**
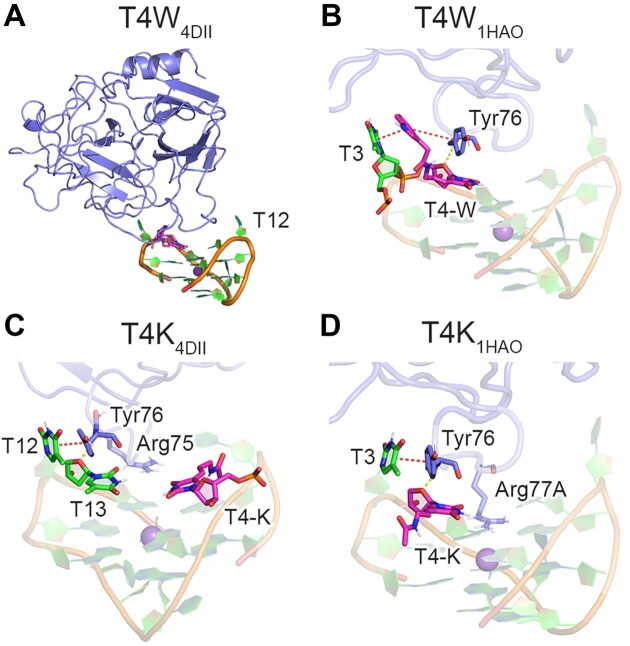
(**A**) Snapshot of T4W_4DII_–thrombin unbinding during simulation. Key interactions across MD simulations on complexes between (**B**) T4W or (C, D) T4K and thrombin initiated from PDB ID: (**C**) 4DII or B, (**D**) 1HAO. π–π interaction (red dotted lines) and hydrogen-bonding (yellow dotted lines) occupancies provided in Supplementary Figures S4, S5 and S8.

**Figure 4.**
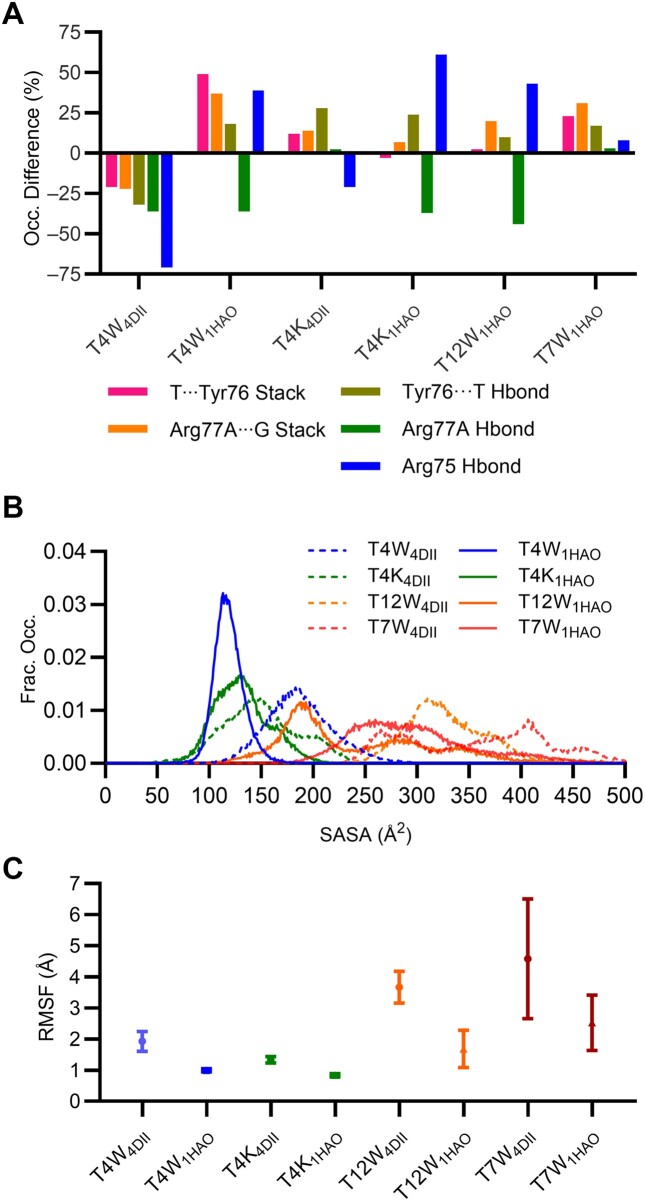
(**A**) Difference in occupancy (%) of key stacking and hydrogen-bonding interactions for each modified TBA relative to canonical TBA in the corresponding thrombin binding orientation. (**B**) Fractional occupancy of the solvent accessible surface area of the modified nucleotides (T4-W, T4-K, T12-W and T7-W) when bound to thrombin according to PDB ID: 4DII (dotted lines) and 1HAO (solid lines). (**C**) RMSF of the modified nucleotides in the two poses of the TBA–thrombin complex.

MD simulations initiated from a high resolution X-ray crystal structure of the aptamer–protein complex (PDB ID: 4DII, Figure 1B, left) (76) highlight key aptamer–protein interactions responsible for binding. Specifically, T12 most frequently π–π stacks with Tyr76, the sugar of neighboring T13 (O4′) persistently hydrogen bonds with the backbone of Tyr76, T3 occupies a shallow hydrophobic pocket on the other side of the TBA–thrombin complex, and several noncovalent interactions periodically exist between the charged side chains of Arg77A and Arg75 and TBA (Figure 2A, Supplementary Figures S4A and S5A). When the T4-W modification is introduced into TBA (Figure 3A, Supplementary Figures S4B, S5A and S6), the bulky and highly flexible W moiety predominantly interacts with the aptamer, displacing Arg75, resulting in less persistent TBA–thrombin interactions involving Tyr76, Arg77A and Arg75 (Figure 4A). In fact, the T12–T13 loop of T4W unbinds from thrombin during the simulations (Figure 3A and Supplementary Figure S7).

Despite reduced interactions between T4W and thrombin when bound according to PDB ID: 4DII contradicting the experimentally-reported 2.8-fold enhanced thrombin affinity (Table 1) (31), a second binding configuration that has a similar binding affinity was identified using Cytolysin A (ClyA) biological nanopores (53). The second complex was proposed to differ from the binding configuration discussed above (PDB ID: 4DII) (76) by a ∼180° rotation about the principal axis of TBA (PDB ID: 1HAO (84), Figure 1B). In the TBA–thrombin 1HAO crystal structure, T12 and T3 exchange binding positions relative to 4DII, with T12 now occupying the shallow hydrophobic pocket and T3 π–π stacking with Tyr76. The same two TBA binding poses can be found in crystal structures of thrombin with TBA bound at exosite I and the larger HD22 aptamer bound at exosite II (PDB IDs: 5EW1 and 5EW2) (97). Interestingly, the introduction of an abasic site (tetrahydrofuran or THF) or adenine at T3 or T12 impacts the ratio of the two isomers as measured using the ClyA nanopores (53), with the discrepancy for the abasic site rationalized based on evidence the T3 variant binds as per the 1HAO pose (PDB ID: 4LZ4), while the T12 counterpart binds as 4DII (PDB ID: 4LZ1) (71). Furthermore, X-ray crystal structures of TBA containing lactose, glucose, or leucine bound to a triazole moiety at N3 of T3 (PDB IDs: 6Z8V, 6Z8W and 6Z8X) (32) show the modified nucleotides bound in a hydrophobic region of thrombin are similar to that encapsulating T3 in the 1HAO crystal structure (84).

MD simulations of the canonical TBA–thrombin complex initiated from the 1HAO crystal structure show similar stabilizing aptamer–protein interactions as the 4DII binding pose, further emphasizing the feasibility of this second, infrequently discussed binding orientation. Specifically, T3 π–π stacks with Tyr76, T4 persistently hydrogen-bonds with the Tyr76 backbone, T12 occupies the hydrophobic pocket on the other side of the complex, and Arg75 and Arg77A continue to interact with several TBA residues (Figure 2B, Supplementary Figures S5B and S8A). While T12 maintains interactions with the thrombin hydrophobic pocket upon incorporation of T-W at T4 of TBA (Supplementary Figures S5B and S8B–S9), the indole moiety of W is sandwiched between T3 and Tyr76 for 100% of the simulation time (Figure 3B and Supplementary Figure S8B). In fact, the T3 binding pocket in the 1HAO pose easily expands to accommodate this extended π–π stacked system, which contrasts the inability of the W moiety to fit in the T3 binding pocket in the 4DII pose. The frequency of the neighboring T4···Tyr76 hydrogen bond and Arg77A π–π stacking and hydrogen-bonding interactions increase, while the prevalence of the hydrogen-bonding interactions with Arg75 decrease (Figure 4A). The strong preference for T4W binding to thrombin in the 1HAO over 4DII binding pose is further demonstrated by decreased solvent accessibility and flexibility of the probe (Figure 4B, C, Supplementary Figures S6, S9 and S10 and Supplementary Table S7).

Collectively, the enhanced TBA–thrombin interactions observed in the 1HAO binding pose compared to canonical TBA rationalize the experimentally-observed increase in binding affinity upon T4 modification (Table 1) (31). In fact, the predicted favored T4W_1HAO_ binding orientation from simulations initiated from a crystal structure of the canonical TBA–thrombin complex is in excellent agreement with crystallographic data (PDB ID: 6EO6) (31), validating our computational approach. In addition, although previous proposals suggest that the difference in stacking at T3 explains the observed enhanced binding (31), our simulations demonstrate that interactions between the W moiety, neighboring TBA nucleotides, and thrombin residues lead to cascading effects that affect several key contacts in the aptamer–protein complex (Figure 4A).

### T4K enhances TBA interactions with thrombin regardless of the binding orientation, rationalizing the observed greater binding affinity than T4W

Although T4-K lacks the terminal indole ring of T4-W (Figure 1C) that drives enhanced thrombin binding affinity compared to canonical T4, an even larger, 7-fold enhancement in TBA binding to thrombin was reported upon incorporation of T-K at T4 (Table 1) (31). When T4K was modeled bound to thrombin as per the 4DII X-ray crystal structure, the peptide linkage of K does not form long-lasting intermolecular interactions with thrombin (Figure 3C, Supplementary Figures S5A and S12A). Nevertheless, T12···Tyr76 π–π stacking, T13···Tyr76 hydrogen bonding, and Arg77A interactions intensify (Figure 4A and Supplementary Figure S12A). This favorable T4K_4DII_ binding mode contrasts the disruption observed in T4W_4DII_, highlighting how the smaller K modification can be readily accommodated at T4. Furthermore, thrombin binding of T4K remains favorable in the 1HAO pose (Figure 3D, Supplementary Figures S5B and S12B). Indeed, T3 continues to π–π stack favorably with Tyr76, while the T4···Tyr76, Arg77A···G stacking and Arg75 hydrogen-bonding interactions are more persistent for T4K than unmodified TBA (Figure 4A). A new hydrogen-bonding interaction also arises between Asn78 and T12 (Supplementary Figure S12A). Thus, in contrast to T-W, the smaller size of T-K allows T4K to bind to thrombin in two relative orientations, with both poses resulting in similar modification solvent shielding and flexibility (Figure 4B, C).

The enhanced thrombin binding of T4K compared to T4W was previously attributed to an energetic cost of T4W binding due to differences in the backbone conformation at T3 in the T4W–thrombin complex, which significantly displaces T3 (PDB ID: 6EO6 (31); Supplementary Figure S13). However, our simulations reveal that the structural dynamics at T3 is consistent for canonical TBA, T4W and T4K bound to thrombin (Supplementary Figure S13). This is further supported by simulations initiated from the crystal structure of the T4W– or T4K–thrombin complex (31), which are in excellent agreement with our simulations (Supplementary Figure S11). Although an additional hydrogen bond was proposed to form between K and Arg77A (3.7 Å separation in X-ray crystal structure) (31), our simulations verify the weak nature of this interaction (1.8% occupancy, Supplementary Figure S5B). Instead, our dynamic structural data suggests the enhancement in key TBA–thrombin interactions in both aptamer–protein binding poses rationalizes the improved binding with respect to canonical TBA and the observed larger increase in thrombin binding affinity for T4K (7.0-fold) compared to T4W (2.8-fold, Table 1) (31).

### While T12W strengthens thrombin binding through interactions with the 70S loop, simultaneous W incorporation at T4 and T12 also results in an arginine cage that synergistically enhances binding

**Figure 5.**
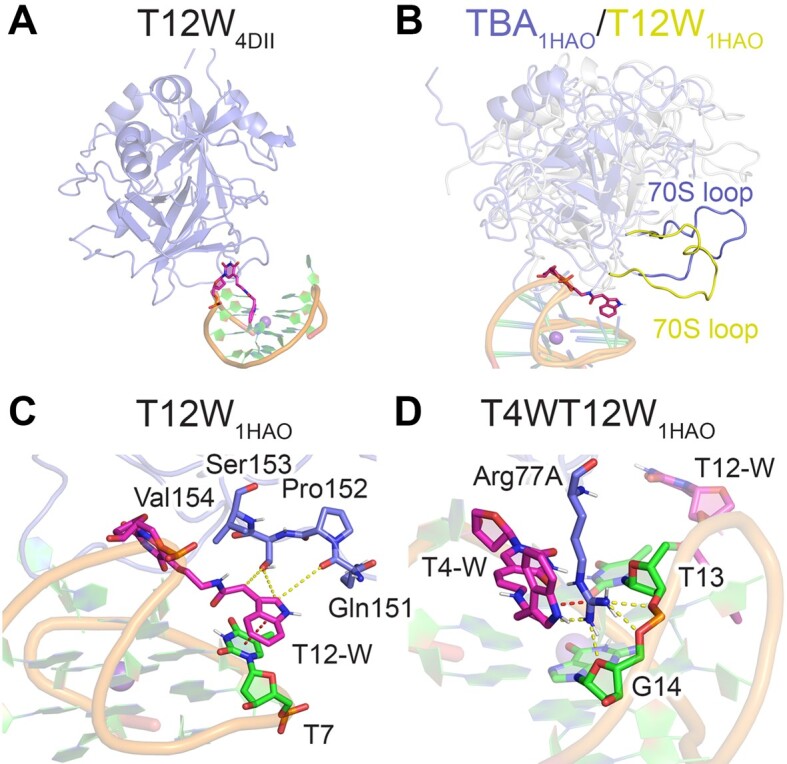
(**A**) Snapshots of T12W_4DII_–thrombin unbinding during simulation. (**B**) Overlay of MD representative structures for TBA_1HAO_–thrombin (blue) and T12W_1HAO_–thrombin (yellow), highlighting different relative orientations of the aptamer and 70S loop of thrombin. Key interactions across MD simulations on the (**C**) T12W_1HAO_–thrombin complex, highlighting T12-W interactions with the 70S loop, or (**D**) T4WT12W_1HAO_–thrombin complex, highlighting the Arg77A cage. Additional π–π interaction (red dotted lines) and hydrogen-bonding (yellow dotted lines) occupancies in Supplementary Figures S14, S15 and S18.

After T4W, T12W leads to the largest (2.2-fold) increase in canonical TBA–thrombin binding (Table 1) (31). When simulations of the T12W–thrombin complex were initiated from the 4DII binding orientation, the T-W nucleotide is even more dynamic and solvent exposed at T12 than T4 (Figure 4B–C), and thrombin becomes unbound from the T3–T4 loop (Figure 5A and Supplementary Figure S16), suggesting this binding pose is not favorable. In contrast, when simulations are initiated from the 1HAO TBA–thrombin complex, the T12-W nucleobase occupies the thrombin hydrophobic pocket, while the 70S loop is recruited to interact with the W moiety (Figure 5B, Supplementary Figures S14B–S15B). While previous work theorized that T12-W could reach Arg75, Ser72, Ser153 and Val154 (31), our simulations demonstrate that the 70S loop changes position to interact with the modification (Figure 5B). In fact, the indole ring of T12-W is solvent shielded (Figure 4B) and the modified nucleotide is less flexible (Figure 4C), sandwiched between T7 and the 70S loop (Figure 5C). While the stacking between T3 and Tyr76 observed in the canonical TBA–thrombin complex are not disrupted upon T12 modification, the T4···Tyr76 hydrogen bond and Arg77A···G stacking interactions are marginally more persistent, while Arg75 hydrogen bonding has significantly increased (Figure 4A). The interactions between T12-W and the 70S loop rationalize the experimentally-observed increased binding stability relative to canonical TBA (31). Nevertheless, T12W binds more weakly than T4W to thrombin since T4-W persistently stacks between T3 and Tyr76 (Supplementary Figure S8B), which also significantly strengthens T4···Tyr76 hydrogen bonding, Arg77A···G stacking, and Arg75 hydrogen bonding (Figure 4A). Additionally, T-W is more solvent exposed and flexible at T12 compared to T4 (Figures 4B, C).

When W is simultaneously incorporated into the T4 and T12 positions of TBA, the thrombin binding pose found in the 4DII crystal structure is not favored, with the protein unbinding from the T3–T4 loop (Supplementary Figure S17). This is not surprising as a single T-W at either position is not favorable in this aptamer–protein binding orientation. In contrast, when T4WT12W simulations are initiated from PDB ID: 1HAO (84), a stable modified TBA–thrombin complex is observed. Specifically, T12-W behaves highly similar in the presence and absence of T-W at T4, with the W moiety at T12 sandwiched between the 70S loop of thrombin and T7 (Figure 5D, Supplementary Figures S15B and S18B). However, unlike T4W, the indole ring of W at T4 does not remain intercalated between T3 and Tyr76 in the doubly-modified system, even when simulations are initiated from this starting orientation. Instead, T4-W aids formation of an ‘Arg77A cage’ that places Arg77A between the indole ring of W, T13, and G14 nucleobases, and the backbone of T13–G14 (Figure 5D). In fact, the two modified TT loops work together to grasp thrombin in the region of Arg77A. As Arg77A cage formation does not occur in the presence of either single modification, this demonstrates the synergistic nature of the W adducts at T4 and T12, and rationalizes the greatest binding affinity for the doubly-modified T4WT12W aptamer, which is 3.3–4.2 fold greater than for either singly-modified TBA (i.e. T4W and T12W) and 9.3-fold greater than canonical TBA (Table 1) (31).

### Despite being distant from the TBA–thrombin binding interface, W at T7 interacts with thrombin to strengthen binding and further enhance thrombin interactions with W at T4

**Figure 6.**
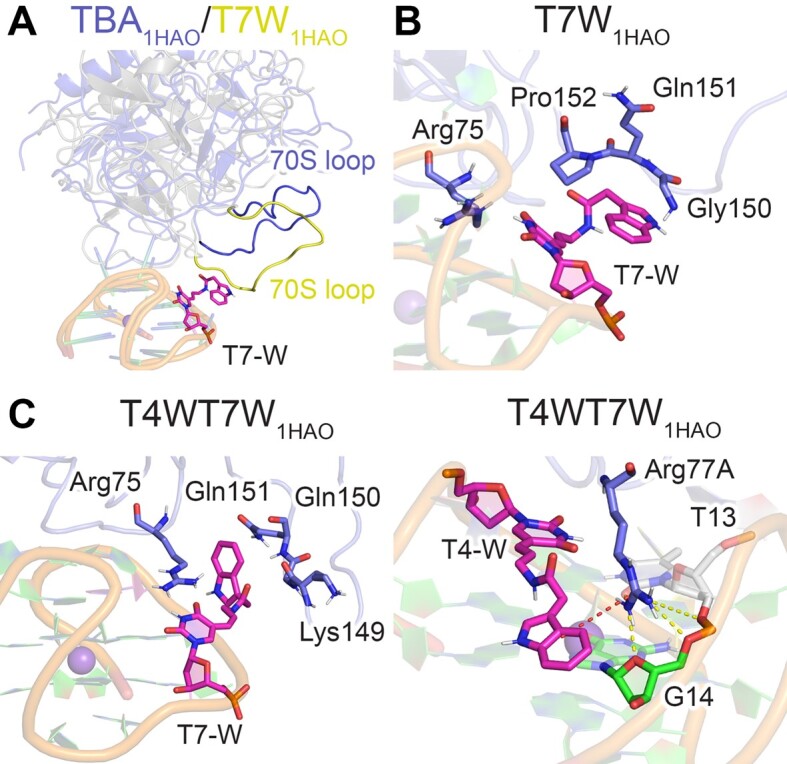
(**A**) Overlay of MD representative structures TBA_1HAO_–thrombin (blue) or T7W_1HAO_–thrombin (yellow), highlighting different relative orientations of the aptamer and 70S loop of thrombin. (**B**) Key interactions across MD simulations on the T7W_1HAO_–thrombin complex. (**C**) Key interactions across MD simulations of the T4WT7W_1HAO_–thrombin complex, highlighting the T7-W interactions with the 70S loop (left) and the Arg77A cage (right). Additional π–π interaction occupancies (red dotted lines) provided in Supplementary Figures S20 and S23, and hydrogen-bonding occupancies (yellow dotted lines) in Supplementary Figure S21.

T7W has been shown to enhance (1.7-fold) TBA binding to thrombin despite T7 falling on the opposite side of TBA than the TT loops responsible for exosite I binding (Table 1) (31). When simulations of the T7W–thrombin complex were initiated from the 4DII crystal structure, T-W is highly flexible (Figure 4C) and thrombin unbinds at the T3–T4 loop (Supplementary Figure S19). In contrast, T7-W is significantly less dynamic in the 1HAO binding pose (Figure 4C). In fact, T7-W folds against TBA such that the linker extends the indole moiety toward thrombin (Figure 6A). Although previous work proposed that T-W at T7 could reach Arg77, Ser72, Ser153 and Val154 (31), our simulations show that the modification recruits the 70S loop to enhance binding in a similar manner as discussed for T12W (Figure 6A, B). T3···Tyr76 π–π stacking, T4···Tyr76 hydrogen bonding, as well as Arg77A and Arg75 noncovalent interactions are also enhanced compared to canonical TBA (Figure 4A, Supplementary Figures S20B and S21B). The cumulation of these improved interactions explains the greater thrombin binding affinity for T7W over TBA (31). Nevertheless, the T7W–thrombin interactions are less persistent (Figure 4A), and T7-W has increased solvent exposure and flexibility compared to T12-W (Figure 4B, C), which correlates with the slightly smaller thrombin affinity of T7W relative to T12W (31).

Although incorporation of T-W at T4, T7, or simultaneously at both positions of the TBA–thrombin complex in the 4DII binding orientation leads to protein unbinding (Supplementary Figures S7, S19 and S22), incorporation of T-W at both T4 and T7 further fine-tunes TBA–thrombin interactions in the 1HAO pose (Figure 6C, Supplementary Figures S21B–S23B). Specifically, as seen for singly-modified T7W, W at T7 in T4WT7W extends towards thrombin and recruits the 70S loop to facilitate binding (Figure 6C). Furthermore, as discussed for T4WT12W, T4-W is unable to intercalate between T3 and Tyr76, even when simulations were initiated from a pre-configured structure. Instead, in the presence of T7-W, T4-W facilitates formation of an Arg77A cage that encases Arg77A between W, G14, T13 and the T13–G14 backbone (Figure 6C). Nevertheless, this Arg77A cage is less persistent than that observed for T4WT12W (Figures 5D and 6C). Thus, the Arg77A cage formed in the presence of T4-W coupled with interactions between T7-W and the 70S loop rationalize the experimentally-observed synergistic impact of T4-W and T7-W (8.7-fold), yet smaller combined effect than T4-W and T12-W (9.3-fold, Table 1) (31).

### Despite apparent symmetry of TBA TT loops, the relative proximity of T-W to the TGT loop results in differential behavior at pseudosymmetrically related sites

**Figure 7.**
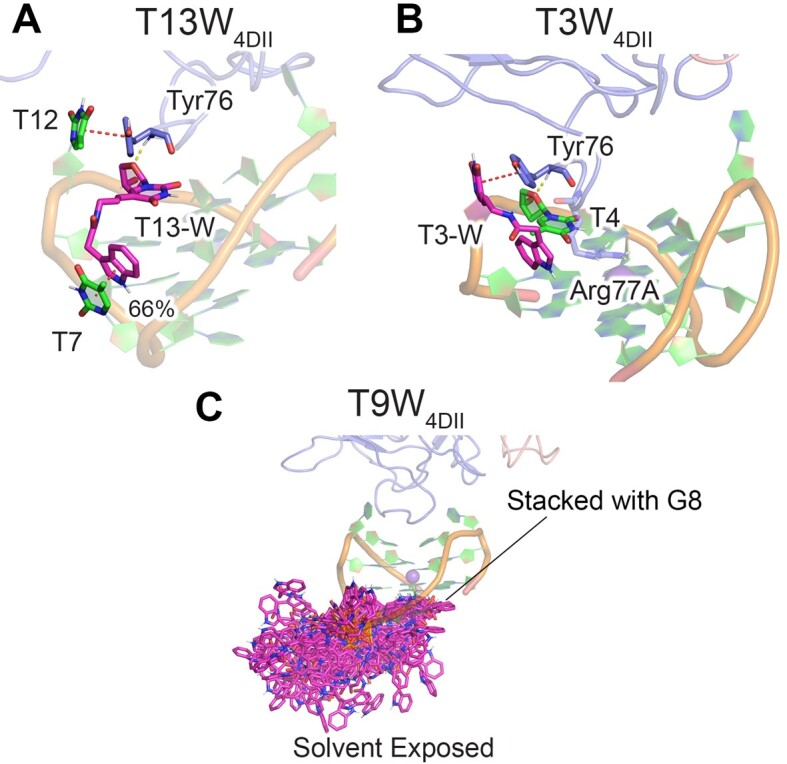
Key interactions across MD simulations from PDB ID: 4DII on the (**A**) T13W_4DII_–thrombin and (**B**) T3W_4DII_–thrombin complexes. (**C**) Overlay of T9-W conformations from T9W_4DII_–thrombin simulations (100 frames total). Additional π–π (red dotted lines) and hydrogen-bonding (yellow dotted lines) occupancies provided in Supplementary Figures S24 and S25.

The 4DII and 1HAO X-ray crystal structures of canonical TBA–thrombin highlight the symmetry of the TT loops (Figure 1B). Specifically, T3 or T12 are interchangeable (stack with Tyr76 or occupy a hydrophobic pocket) and T4 or T13 are pseudosymmetrically related sites (hydrogen bond with the Tyr76 backbone), while T7 and T9 appear on opposite sides of the symmetric TGT loop. It is therefore intriguing that T3W and T13W have reduced thrombin binding affinity compared to canonical TBA (Table 1), despite the symmetry-related T12W and T4W modifications enhancing TBA binding (31). To better understand this differential behaviour, the preferred structure of the T4W, T12W, and T7W–thrombin complexes (1HAO binding mode) were compared to the corresponding T13W, T3W, and T9W–thrombin complexes (4DII binding mode). Despite the indole ring of T-W at T4 being sandwiched between T3 and Tyr76 in T4W_1HAO_ (Figure 3B), the indole ring of T13-W stacks with T7 (Figure 7A and Supplementary Figure S24A). The resulting conformational change reduces thrombin interactions with T12 as well as native TBA–Arg75/Arg77A contacts (Figure 4A and Supplementary Figures S24A–S25A), rationalizing the observed reduced target binding affinity (0.5-fold compared to canonical TBA, Table 1) (31). It is important to note that the difference in the behaviour of T13-W and T4-W arises at least in part due to the location and tilt of the TGT loop as well as the C5 position of T13, which permits close contact of the W moiety and the TGT loop for T13-W, but not the T4-W symmetric partner. Similarly, T12-W is sandwiched between T7 and the 70S loop of thrombin (Figure 5C), while T3-W is spatially removed from the TGT loop, and therefore cannot be supported to recruit the 70S loop. Instead, the W moiety of T3-W is directed towards the edges of the GQ as previously speculated (31) and T3 interactions with the thrombin hydrophobic pocket are significantly reduced (Figure 7B and Supplementary Figures S24B–S25B), explaining the decreased thrombin affinity of T3W compared to canonical TBA and T12W (Table 1) (31). Finally, although T7 and T9 appear on opposing ends of the TGT loop, the 3′-to-5′ directionality of the DNA backbone permits T7-W to fold against the aptamer to recruit the 70S loop (Figure 6A), while T9-W is highly dynamic (Supplementary Table S7), being either solvent exposed or stacked with G8 (Figure 7C). This inherent flexibility reduces interactions between the probe and thrombin (Supplementary Figures S24C–S25C), rationalizing the observed low thrombin affinity of T9W (0.5-fold compared to canonical TBA, Table 1) (31). Overall, these three comparisons (T4 versus T13, T3 versus T12, and T7 versus T9) underscore that chemical modifications differentially behave in pseudosymmetric TBA sites due to the influence of the TGT loop.

## Discussion

Although chemical modification of nucleotides has been proven to enhance the function of aptamers by increasing nucleic acid stability and strengthening target binding (12–15,17,18,20,22–26,30–82), little is understood about the impact of modification type, position and number on aptamer performance. An atomic level appreciation of the influence of nucleotide chemical composition on aptamer structure is critical for optimizing the design of new and improved nucleic acids for a host of applications in medicine and biotechnology. In this light, the classic 15-mer TBA has been used to gain information about chemical modifications due to its small size and the availability of high-resolution crystal structures of the TBA–thrombin binding interface (76,84). Indeed, a wealth of experimental studies have incorporated uniquely synthesized modifications into different positions of TBA and monitored changes in aptamer melting temperature, thrombin binding, and/or pro-thrombin time (12,18,20,22,25,30–65). Despite the abundance of experimental studies on the chemical properties of modified TBA, little experimental data is available for the structure of modified TBA–thrombin complexes (31,32,71,78) at least in part due to the challenging, costly, laborious, and time-consuming process of solving X-ray crystallography or cryo-EM structures.

To complement experimental data on modified TBA, previous computer modeling has provided information about noncovalent interactions with thrombin at the modification site, generally using single, short timescale MD simulations for only the best performing aptamer (22,23,25,35–37,39,42,47,49,52,57–59,65,69). In addition to falling short of best practices regarding simulation procedures necessary to achieve statistically reliable descriptions of nucleic acid structure, which requires long timescales and replicates (91–93), such studies typically do not analyze the impact of modifications on pre-existing canonical TBA interactions with thrombin, and therefore, the holistic effects of modifications are not clear. Furthermore, many studies that couple traditional experiments with computer modeling have focused on G modifications (12,22–25,35–37,39,43,45,47,49,52,57–59,66–70), with fewer computational studies examining T modifications (22,25,30–32,34,38,42–45,58–60,65,66,69,71,72), which are particularly relevant as the TT loops of TBA directly interact with exosite I of thrombin upon binding (76,84). Furthermore, modifications introduced at the TT loops typically represent small chemical changes to the canonical nucleotide (30,44,45,58,60,66), often to the backbone (25,38,42,43,45,59,60,65,69,72). These works also generally focus on a single T modification site in TBA. Indeed, to the best of our knowledge, only one previous study has provided structural information for a T modification (C5 methyl group replaced with a furan moiety) incorporated at six different aptamer positions (66), which underscored the valuable insight obtained about aptamer design by studying both modifications that do and those that do not enhance aptamer function. Furthermore, only a single study has considered the simultaneous effects of T modifications (methylene-carboxyl moiety at C5) at two TBA sites (22). Finally, previous computational studies have typically considered only a single aptamer–target binding pose. Thus, while previous computational work has afforded some useful information about the influence of modification type on aptamer–protein binding, the small size of the modifications investigated to date, the lack of diversity in modification sites considered, the scarcity of information about the interplay between multiple modifications, and consideration of single binding orientations leaves many questions about the behavior of potential modification designs unanswered. Therefore, to complement previous literature, the present work used computational methods to uncover the dynamical origin of differential thrombin binding affinity for a family of modified TBAs that contain a large, flexible C5-modified T nucleotide at different sequence locations (Figure 1 and Table 1). Our simulations have provided detailed structural information that has uncovered important themes that can be used to guide future aptamer design.

### Bulky chemical modifications can influence the native aptamer–target binding orientation and/or induce conformational changes in the target to enhance binding

Although canonical TBA has been shown to bind to thrombin in two isomeric configurations that have similar binding affinities (Figure 1B) (53,76,84), the flexibility of the W moiety at C5 of T4, T7 or T12 renders the binding orientation observed in PDB ID: 4DII unstable, with thrombin unbinding from the aptamer at the TT loop opposite the modification site (e.g. unbinding at T12–T13 for T4W, but unbinding at T3–T4 for T12W, Figures 3A and 5A). Nevertheless, the TBA–thrombin binding orientation in PDB ID: 1HAO can readily accommodate T-W at T4, T7 or T12, with the modification differentially fine-tuning interactions with the protein target depending on modification site. Among the TBA modification positions experimentally-observed to enhance thrombin binding (Table 1) (31), incorporation of W at T4 increases stacking with key protein binding residues (e.g. Tyr76), which augments neighboring interactions (e.g. hydrogen bonding with Arg75 side chain and Tyr76 backbone, Figure 4). In contrast, the long indole–nucleobase linker of W permits the modification at T12 and T7 to recruit the 70S loop of thrombin (Figures 5 and 6) and thereby increase aptamer affinity, albeit to a lesser extent than W at T4 (Table 1).

The experimentally-observed enhancement in thrombin affinity for T-W at T4 or T12 (31) that is rationalized by the present work is unique compared to other modifications incorporated at these sites, which are smaller chemical alterations, often to the backbone, and tend to negatively affect target binding (25,31,42,61,62). Furthermore, the impact of T7 modification is particularly interesting, highlighting that incorporation of a large (long) modification can strengthen aptamer binding through direct target interactions despite being distal to the canonical aptamer binding location, without changing the aptamer binding orientation (i.e. contacts between TT loops and thrombin are maintained). Indeed, other experimental studies have reported increased thrombin binding affinities when large chemical groups are added to T7 (31,32,49,58,82). In contrast, smaller modifications at T7 marginally impact or decrease aptamer performance (30,34,38,42–45,60,66,69,75), with the exception of enhanced TBA-thrombin affinity for unlocked nucleic acid (UNA) analogues (33,60). Thus, modifications to nucleic acids sites both close to and removed from target binding locations must be carefully considered in aptamer design.

### Structural motifs spatially removed from the target binding site can result in differential effects of modified nucleotides at pseudosymmetrical aptamer positions

Despite the pseudosymmetry of the TBA TT loops, T-W was reported to exhibit differential behavior when incorporated at T3 and T13 compared to the symmetry-related T12 and T4 partners (Table 1) (31). Other experimental studies have also reported similar phenomena (31,32,42,49,82). For example, lactose, glucose, or leucine-derived modifications at T3 result in enhanced thrombin binding, while analogous T12 modifications reduce target affinity (32). In the case of T-W, our simulations suggest this differential behavior arises because the remainder of the aptamer must stabilize the location of the long, flexible W moiety, with the local modification geometry being heavily influenced by the proximity and 3′-to-5′ directionality of the TGT loop. Specifically, while T12-W is supported by T7 in the TGT loop, the distal location of T3 from the loop results in T3-W interactions with the edge of the GQs rather than increasing interactions with thrombin (Figures 5 and 7). Alternatively, although T4-W forms strong interactions with thrombin due to stacking with T3, T12 is unable to offer the same stabilization to T13-W, resulting in T13-W interactions with the TGT loop (Figures 3 and 7), which is closer to the C5 moiety of T13 than T4. Interestingly, a break in TBA pseudosymmetry generally arises for large modifications (31,32,49,82), while smaller chemical changes result in similar behavior when incorporated into symmetrically-equivalent TT loop positions (38,44,45,60,61,66,75,78). For example, modification of T3 or T12 by replacing oxygen with sulfur at C4 (60), substituting methyl with fluorine (44) or a methylene-carboxyl group at C5 (22), introducing an abasic site (71), or converting T to L-isothymidine (L-isoT) (42) has a nearly identical impact on target binding affinity. The structural rationalization of the experimentally-observed TBA asymmetry uncovered by our work showcases that future aptamer design must carefully consider how nucleic acid structural motifs assumed to be removed from the target binding site may influence the behavior of the modification.

Although T7 and T9 appear on opposite ends of the TGT loop, modifications at these sites also differentially impact thrombin binding because the 3′-to-5′ directionality tilts the loop with respect to the GQs and prevents T9-W from reaching the 70S loop as seen for T7-W (Figures 6 and 7). Experimental work on other modifications has also highlighted the asymmetry of the T7 and T9 positions (39,43,44,60,61). For example, the UNA (60) and 3′-inverted nucleotide modifications (43) more significantly enhance aptamer function at T7 than T9, while L-isoT more noticeably improves TBA performance at T9 compared to T7 (42). This reinforces the importance of understanding the three-dimensional structure of modified nucleic acids, as well as considering the ability of modifications in structural motifs removed from canonical binding sites to directly interact with the target.

### Decreasing the size of chemical modifications can enhance target affinity by fostering multiple aptamer binding orientations

Consideration of the T-K modification, which lacks the indole group of T-W (Figure 1C), raises several important design principles for chemically-modified nucleic acids. Despite the indole moiety of T4-W playing a critical role in enhanced thrombin affinity, truncation of the bulky moiety results in the greatest enhancement in TBA performance (Table 1) (31), which simulations reveal is likely due to augmented interactions in two different modified TBA–thrombin binding orientations (Figures 3C, D and 4). This entropic argument for enhanced binding is unique among computational studies, which typically consider only one TBA–thrombin binding pose (i.e. PDB ID: 4DII (22,23,25,35,36,43,45,49,63–64,66,71,74,89) or 1HAO (38,39,49,52,57,58,68). Indeed, only one previous study initiated modeling from multiple aptamer–protein binding orientations (22), although some binding poses considered do not represent 1:1 aptamer–protein complexes and the differences between binding poses were not critically analyzed to understand the impact of chemical modifications on thrombin affinity. The dependence of binding orientation on the chemical composition of the modification underscored by our work suggests that modified TBAs previously investigated in the literature may bind to thrombin in more than one way. It is important to note that the ability to adopt different binding modes is closely related to the pseudosymmetry of the TT loops. Indeed, many, generally smaller, modifications exhibit similar behavior when incorporated at T3 and T12 (38,44,45,60,61,66,71,75). Moreover, in the case of an abasic site, the similar thrombin affinity at T3 or T12 can be rationalized based on evidence the T3 variant binds as per the 1HAO pose (PDB ID: 4LZ4), while the T12 counterpart binds as 4D11 (PDB ID: 4LZ1) (71). Thus, our simulations further highlight the impact of modification size on aptamer function. To more thoroughly understand how modification type influences thrombin binding, the structural implications of a wider range of modifications in different binding poses must be thoroughly investigated in the future.

### Multiple modifications can generate new structural motifs that improve target binding

Compared to singly-modified aptamers, fewer TBA molecules that contain more than one modification have been synthesized (17,22,31,42,61). Among these systems, several examples demonstrate that the binding affinity of singly-modified TBA can be further enhanced by the addition of a second modification (17,22,42). However, few structural explanations are available due to the absence of solved crystal structures. Only one previous computational study used simulated annealing coupled with molecular mechanics calculations to uncover the loss of some and gain of other aptamer–target hydrogen bonds for double methylene-carboxylate modifications at C5 of T compared to singly-modified or canonical TBA, rationalizing the equivalent binding affinity regardless of the level of substitution (22). Our simulations reveal that incorporation of a second modification can fine-tune aptamer–target interactions by working together to generate novel nucleic acid–protein structural motifs. Specifically, two T-W modifications synergistically collaborate to form a cage that can capture key arginine residues of thrombin and thereby enhance target binding (Figures 5 and 6; Table 1). Nevertheless, some experimental studies have shown that two modifications decrease target binding affinity (17,31,42,61). For example, L-isoT at either T3 or T12 enhances thrombin binding, while simultaneous modification of T3 and T12 significantly reduces target affinity (42). The ability to predict such interplays between modifications *de novo* is challenging, further underscoring the importance of the atomic-level data obtained from computational analyses.

## Conclusion

The present work uses MD simulations to reveal the dynamical origin of differential thrombin binding affinity for a series of TBAs containing different sizes, positions and numbers of large, flexible C5-modified T nucleotides. By carefully analyzing multiple TBA–thrombin binding orientations, structural rationalizations were provided for the experimentally-observed aptamer performance (31) and several important aptamer design trends have become apparent, showcasing the advantages of a computational approach. Notably, bulky chemical modifications can alter native aptamer–target binding orientations and/or induce conformational changes in the target protein to facilitate binding, while truncated modifications can further enhance binding despite a smaller size by promoting more than one aptamer–target binding pose. Furthermore, addition of large, flexible modifications to nucleic acid sites either close to or removed from the target binding location can enhance aptamer performance by increasing contacts with the target at the modified nucleotide as well as by more broadly driving the formation of networks of strengthened contacts. Nucleic acid structural motifs generated through aptamer folding (e.g. nucleotide loops) can also play a critical role in the behavior of modified aptamers, differentially interacting with pseudosymmetric modification sites depending on proximity and 5′-to-3′ directionality. Finally, multiple modifications can fine-tune aptamer–target interactions by working together to generate novel nucleic acid–protein structural motifs. Overall, our work underscores the importance of understanding the structural dynamics of diverse sets of modified nucleic acids and the usefulness of computer simulations in obtaining this information. The detailed structure–function relationships of chemically-modified aptamers obtained from the present work will prove invaluable for the rational design of improved nucleic acids for a host of applications in medicine and biotechnology.

## Supplementary Material

gkae729_Supplemental_Files

## Data Availability

The parameter files for modified nucleotides as well as the geometries of dominant conformations for each TBA–thrombin complex are available in the Supplementary Data (Supporting Information Data.zip). Starting structures, modified nucleobase parameters (W and K), and analysis scripts are available through Github (https://github.com/wetmore-lab/2024-NAR-murray-wetmore/) and Zenodo (https://doi.org/10.5281/zenodo.13257325). Full trajectories are available upon request.
