# Supplementary material for: Unlocking precision in aptamer engineering: a case study of the thrombin binding aptamer illustrates why modification size, quantity, and position matter
Source: Nucleic Acids Res. 2024 Sep 1;52(18):10823–35. doi: 10.1093/nar/gkae729 (PMC11472061; doi:10.1093/nar/gkae729)
Supplement: gkae729_Supplemental_Files [file gkae729_supplemental_files.zip › supplementary material_Aug28_FINAL_Corrected.pdf]

Unlocking Precision in Aptamer Engineering: A Case Study of the Thrombin Binding Aptamer Illustrates Why Modification Size, Quantity, and Position Matter

Makay M. Murray and Stacey D. Wetmore*

Department of Chemistry and Biochemistry, University of Lethbridge, 4401 University Drive West, Lethbridge, Alberta, Canada, T1K 3M4

Supporting Information

(28 pages)

Contents

Computational Methods S4

Model Building S4

MD Simulation Protocol S4

MD Simulation Analysis S5

Figure S1. Overlays with respect to the thymine nucleobase heavy atoms of all conformers (top) and minimum energy conformers (bottom) isolated in the present work for A) T-W (44) and B) T-K (20). Energy range for conformers is 39.4 kJ·mol^{-1} for T-W and 21.5 kJ·mol^{-1} for T-K. Thymine nucleobase is shown in grey and C5 moiety carbon atoms in magenta S6

Figure S2. A) T-W and B) T-K atomic numbering (top), atom types (middle), and partial charges (bottom) S7

Figure S3. Heavy atom RMSDs of all canonical and modified TBA–thrombin complexes across MD simulation replicas. Replica 1 is highlighted in tan, replica 2 in lavender, and replica 3 in green S8

Figure S4. Key TBA–thrombin interactions observed in MD simulations initiated from PDB ID: 4DII for A) canonical TBA and B) T4W, highlighting contacts with i) Tyr76, ii) hydrophobic pocket, iii) Arg75, and iv) Arg77A. π–π interaction (red dotted lines) and hydrogen-bonding (yellow dotted lines) occupancies provided S9

Figure S5. Occupancies of hydrogen-bonding interactions with Arg75 (magenta) and Arg77A (red) observed in MD simulations initiated from PDB ID: A) 4DII and B) 1HAO, highlighting interactions with canonical TBA (left), T4W (middle), and T4K (right) S9

Figure S6. Overlays of T-W or T-K adducts from MD simulations initiated from PDB ID: 4DII. Overlays consist of 100 frames for each system that were evenly sampled across all replicates S10

Figure S7. Representative snapshots from MD simulations of the T4W–thrombin complex initiated from PDB ID: 4DII, highlighting progression from A) an intact complex to B) unbinding of thrombin at the T12–T13 loop S10

Figure S8. Key TBA–thrombin interactions observed in MD simulations initiated from PDB ID: 1HAO for A) canonical TBA and B) T4W, highlighting contacts with i) Tyr76, ii) hydrophobic pocket, iii) Arg75, and

iv) Arg77A. π–π interaction (red dotted lines) and hydrogen-bonding (yellow dotted lines) occupancies provided S11

Figure S9. Overlays of T-W or T-K adducts from MD production simulation initiated from PDB ID: 1HAO. Overlays consist of 100 frames for each system that were evenly sampled across all replicates S11

Figure S10. Fractional occupancy of solvent accessible surface area (SASA) of T-W/K at various positions in modified TBA–thrombin complexes modeled from PDB ID: A) 4DII and B) 1HAO S12

Figure S11. Overlay of the dominant MD representative structure obtained starting from the canonical TBA–thrombin complex (PDB ID: 1HAO) with A) the X-ray crystal structure or B) the MD representative structure obtained starting from the X-ray crystal structure of the modified TBA–thrombin complex for $T4W_{1HAO}$ (left) and $T4K_{1HAO}$ (right). S13

Figure S12. Key T4K–thrombin interactions observed in MD simulations initiated from PDB ID A) 4DII or B) 1HAO, highlighting contacts with i) Tyr76, ii) hydrophobic pocket, iii) Arg75, and iv) Arg77A. π–π interaction (red dotted lines) and hydrogen-bonding (yellow dotted lines) occupancies provided........ S14

Figure S13. A) Crystal structures of thrombin bound to canonical TBA (PDB ID: 1HAO, left), T4W (PDB ID: 6EO6, center), and T4K (PDB ID: 6EO7, right), highlighting different backbone orientations at T3. B) Overlays of 100-frames for each system that were evenly sampled across all replicates. C) Violin plots of the backbone torsion angles of T3 from MD simulations of thrombin bound to canonical TBA, T4W (superscript W) or T4K (superscript K) initiated from PDB ID: 1HAO S15

Figure S14. Key T12W–thrombin interactions observed in MD simulations initiated from PDB ID A) 4DII or B) 1HAO, highlighting contacts with i) Tyr76, ii) hydrophobic pocket, iii) Arg75, and iv) Arg77A. π–π interaction (red dotted lines) and hydrogen-bonding (yellow dotted lines) occupancies provided........ S16

Figure S15. Occupancies of hydrogen-bonding interactions with Arg75 (magenta) and Arg77A (red) observed in MD simulations initiated from PDB ID: A) 4DII and B) 1HAO, highlighting interactions with canonical T4W (left), T12W (middle), and T4WT12W (right) S16

Figure S16. Representative snapshots from MD simulations of the T12W–thrombin complex initiated from PDB ID: 4DII, highlighting progression from A) an intact complex to B) unbinding of thrombin at the T3–T4 loop S17

Figure S17. Representative snapshots from MD simulations of the T4WT12W–thrombin complex initiated from PDB ID: 4DII, highlighting progression from A) an intact complex to B) unbinding of thrombin at the T3–T4 loop S17

Figure S18 Key T4WT12W–thrombin interactions observed in MD simulations initiated from PDB ID: A) 4DII or B) 1HAO, highlighting contacts with i) Tyr76, ii) hydrophobic pocket, iii) Arg75, and iv) Arg77A. π–π interaction (red dotted lines) and hydrogen-bonding (yellow dotted lines) occupancies provided..... S18

Figure S19. Representative snapshots from MD simulations of the T7W–thrombin complex initiated from PDB ID: 4DII, highlighting progression from A) an intact complex to B) unbinding of thrombin at the T3–T4 loop S18

Figure S20. Key T7W–thrombin interactions observed in MD simulations initiated from PDB ID: A) 4DII or B) 1HAO, highlighting contacts with i) Tyr76, ii) Hydrophobic pocket, iii) Arg75, and iv) Arg77A. π–π interaction (red dotted lines) and hydrogen-bonding (yellow dotted lines) occupancies provided........ S19

Figure S21. Occupancies of hydrogen-bonding interactions with Arg75 (magenta) and Arg77A (red) observed in MD simulations initiated from PDB ID: A) 4DII and B) 1HAO, highlighting interactions with canonical T4W (left), T7W (middle), and T4WT7W (right) S19

Figure S22. Representative snapshots from MD simulations of the T4WT7W–thrombin complex initiated from PDB ID: 4DII, highlighting progression from A) an intact complex to B) unbinding of thrombin at the T12–T13 loop. C) The movement of the 70S loop from TBA_{1HAO} (blue) to $T4WT7W_{1HAO}$ (yellow)........... S20

Figure S23. Key T4WT7W–thrombin interactions observed in MD simulations initiated from PDB ID: A) 4DII or B) 1HAO, highlighting contacts with i) Tyr76, ii) hydrophobic pocket, iii) Arg75, and iv) Arg77A. π–π interaction (red dotted lines) and hydrogen-bonding (yellow dotted lines) occupancies provided..... S20

Figure S24. Key T4WT7W–thrombin interactions observed in MD simulations initiated from PDB ID: 4DII, A) T13W, B) T3W, and C) T9W, highlighting contacts with i) T12 and Tyr76, ii) T3 and a hydrophobic pocket, iii) Arg75, and iv) Arg77A. π–π interaction (red dotted lines) and hydrogen-bonding (yellow dotted lines) occupancies provided S21

Figure S25. Occupancies of hydrogen-bonding interactions with of Arg75 (magenta) and Arg77A (red) observed in MD simulations initiated from PDB ID: 4DII, highlighting interactions with A) T13W, B) T3W, and C) T9W S21

Table S1. Unresolved thrombin residues in X-ray crystal structures of TBA–thrombin complexes that were added during model building S22

Table S2. Summary of aptamers and thrombin binding orientations considered in the present work... S22

Table S3. Number of ions and water molecules added to each aptamer–thrombin model.................. S23

Table S4. Percent occupancy, standard deviation, and standard error for key π–π stacking and hydrogen-bonding interactions in canonical and singly modified aptamers with enhanced binding affinities S24

Table S5. Results from one-way ANOVA for key π–π stacking and hydrogen-bonding interactions grouped across canonical and singly modified aptamers with enhanced binding affinities S24

Table S6. Adjusted P values from Tukey's tests for key aptamer–thrombin π–π stacking and hydrogen-bonding interactions S25

Table S7. RMSF values averaged across simulation replicas for each nucleotide in the aptamer bound to thrombin in the orientation found in the X-ray crystal structures with PDB ID: 4DII and 1HAO. S26

References S27

COMPUTATIONAL METHODS

Model Building

Modified TBA–thrombin complexes were built from X-ray crystal structures of canonical TBA–thrombin complexes (PDB ID: 4DII (2.05 Å)[1] and PDB ID: 1HAO (2.80 Å)[2]). Each crystal structure was initially prepared by removing water, solvent ions, and molecules used for crystallization such as *N*-acetyl-*D*-glucosamine (found only in 4DII) and *D*-phenylalanyl-*N*-[(2*S*,3*S*)-6-[[amino(iminio)methyl]amino]-1-chloro-2-hydroxyhexan-3-yl]-L-prolinamide (found in both 4DII and 1HAO). Unresolved protein residues were manually added using Pymol 2.5 (Table S1),[3] while the unresolved potassium ion in 1HAO was placed equidistant (~2.8 Å) from the eight O6 atoms of the guanine tetrads. Conformational searches were performed for T-W and T-K using the AMBER force field[4] built in Hyperchem Professional 8.0.8[5] (maximum of 10,000 iterations and RMS gradient acceptance = 0.01 $kcal \cdot mol^{-1} \cdot Å^{1}$). The resulting 44 unique conformers for W and 20 for K (Figure S1) were subsequently optimized with B3LYP-D3(BJ)/6-31G(d) using Gaussian 16 (Revision C.01).[6] The minimum energy conformation was aligned with each T residue of TBA, the canonical T was deleted, and the modification conformation adjusted to reduce steric clashes with TBA and/or thrombin where necessary. In our nomenclature, when a thymine residue such as T4 is replaced by T-W the numbered modified thymine is now T4-W, while a TBA molecule in which T4 is replaced with T-W is denoted T4W. The corresponding modified TBA–thrombin complex is denoted $T4W_{PDB\ ID}$ (i.e., $T4W_{4DII}$ is the T4W–thrombin complex in the binding pose present in PDB ID: 4DII). Thrombin residue numbering was used throughout as per PDB ID: 4DII.[1]

Each model was solvated in a truncated-octahedral water box using the TLEaP module of AmberTools18,[7] with a minimum of 10 Å from the solute to the box face in any direction. The systems were neutralized by adding Na^+ counterions, then NaCl was added to bring the models to a physiological salt concentration of 150 mM. Ion counts for each system were determined using the SLTCAP calculator (Number of ions and water molecules added to each aptamer–thrombin model (Table S3)).[8] In total, the canonical and 9 modified TBA–thrombin complexes were modelled from PDB ID: 4D11, while canonical TBA and 6 modified TBA that bound most strongly to thrombin according to the experimental data were also considered in the binding orientation described by PDB ID: 1HAO, resulting in 17 unique systems (Table S3). Appropriate force fields were used to describe DNA (OL15)[9] and the protein (ff14SB),[10] while TIP4P-EW was used for water.[11] Atomic partial charges for T-W and T-K were calculated with RED.v.iii[12] (using the previously identified minimum energy conformers). The antechamber module of AmberTools18[7] was used to assign atom types, which were supplemented by the general amber forcefield (GAFF, Figure S2).[13]

MD Simulation Protocol

All systems were sequentially minimized in four stages, with each stage minimizing a selection of the model. Specifically, the solvent and ions were minimized (1,000 steps of steepest decent (SD) and 3,000 steps of conjugate gradient (CG)), followed by the solute hydrogen atoms (1,000 SD and 1,000 CG), the solute (1,000 SD and 1,000 CG), and finally all atoms (1,000 SD and 3,000 CG). Each system was then brought to a physiological temperature of 310 K through six heating stages from 60 K, using a 25 $kcal \cdot mol^{-1} \cdot Å^{-2}$ restraint on the solute and the Langevin thermostat[14] (1 ps collision frequency). Equilibration was then performed in 5 stages of 10,000 steps, while decreasing the restraints on the solvent from 25 to 20, 15, 10, 5, and 1.5 $kcal \cdot mol^{-1} \cdot Å^{-2}$. The SHAKE[15] algorithm was applied to carbon–hydrogen bonds to enable a timestep of 2 fs. Finally, 1 µs MD production simulations were performed in triplicate (3 µs total) for each model. An NPT ensemble set to 310 K (Langevin thermostat)[14] and 1 bar (Berendsen barostat)[16] was used. The water density was ~1 $g \cdot mL^{-1}$, the periodic boundary condition was enabled, and the non-bonded term cut-off was set to 10 Å. All simulations were run using the pmemd.cuda

program of the Amber18 software suite.[17]

MD Simulation Analysis

Frames were saved every 20 ps for analysis, resulting in 20,000 frames per system over all replicas, which were analyzed using the CPPTRAJ program of AmberTools18.[7] Representative structures were obtained using the cluster function of CPPTRAJ and the hierarchical agglomerative algorithm based on the root-mean-square deviation (RMSD) of all heavy atoms in residues at the TBA–thrombin binding interface (TBA residues T3-G5 and T12-G14; thrombin residues Ala20, Glu23, Ile24, Arg66, His71, Ser72, Thr74, Tyr76, Asn78, Ile79, Glu80, Ile82, Tyr118, and Lys149). The heavy atom RMSD of the protein and TBA backbone over the production simulations was analyzed (Figure S3). In-house scripts were used to evaluate π–π stacking occupancies, with π–π interactions deemed present when the centers of mass of two π systems were ≤ 5 Å and the coplanar angle fell within 0–30° or 150–180°. Hydrogen-bond occupancies were evaluated based on cutoffs for the heavy-atom–to–heavy-atom distance of ≤ 3.4 Å and heavy-atom–hydrogen–heavy-atom angle of ≥ 130°, with hydrogen bond distances measured using the CPPTRAJ hbond command. Three residues (i.e., amino acid sidechains and/or nucleobases) were considered sandwiched when any atom of the middle residue was simultaneously < 4 Å from the two surrounding residues. Block averaging was performed in groups of 100 frames (5 ns) across all replicas for the occupancies of key stacking and hydrogen-bonding interactions to determine averages, standard deviations, and standard errors (Table S4). Datasets were confirmed to be significantly distinct using one-way ANOVA analysis on each metric (Table S5). However, since the degree of freedom was relatively high, a Tukey's test was subsequently performed to verify when key metrics are significantly different or equal (Table S6). Prism software (version 10.2) was used to perform both the one-way ANOVA and the Tukey's tests.

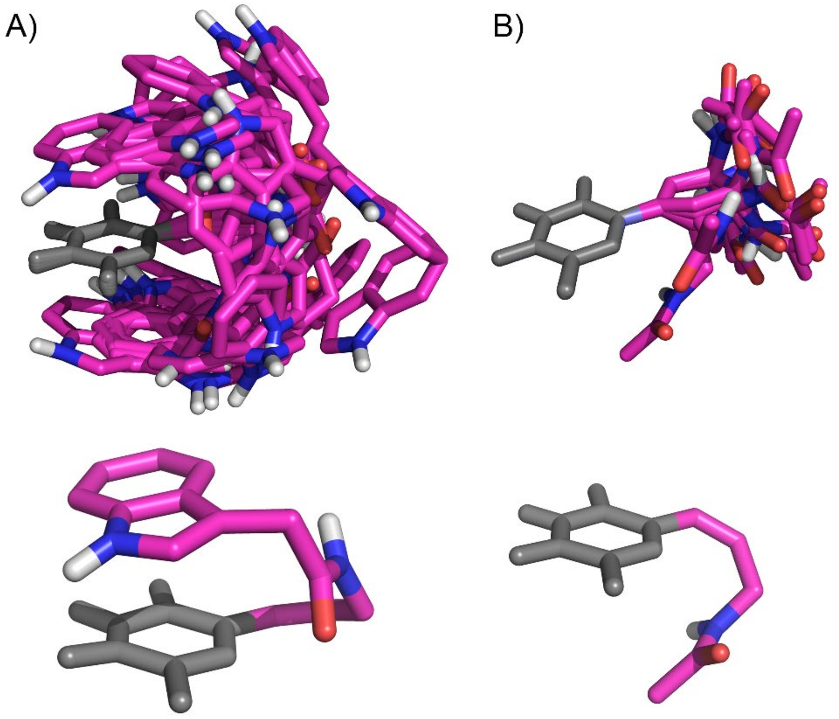

Figure S1. Overlays with respect to the thymine nucleobase heavy atoms of all conformers (top) and minimum energy conformers (bottom) isolated in the present work for A) T-W (44 total) and B) T-K (20 total). Energy range for conformers is 39.4 kJ·mol^{-1} for T-W and 21.5 kJ·mol^{-1} for T-K. Thymine nucleobase is shown in grey and C5 moiety carbon atoms in magenta.

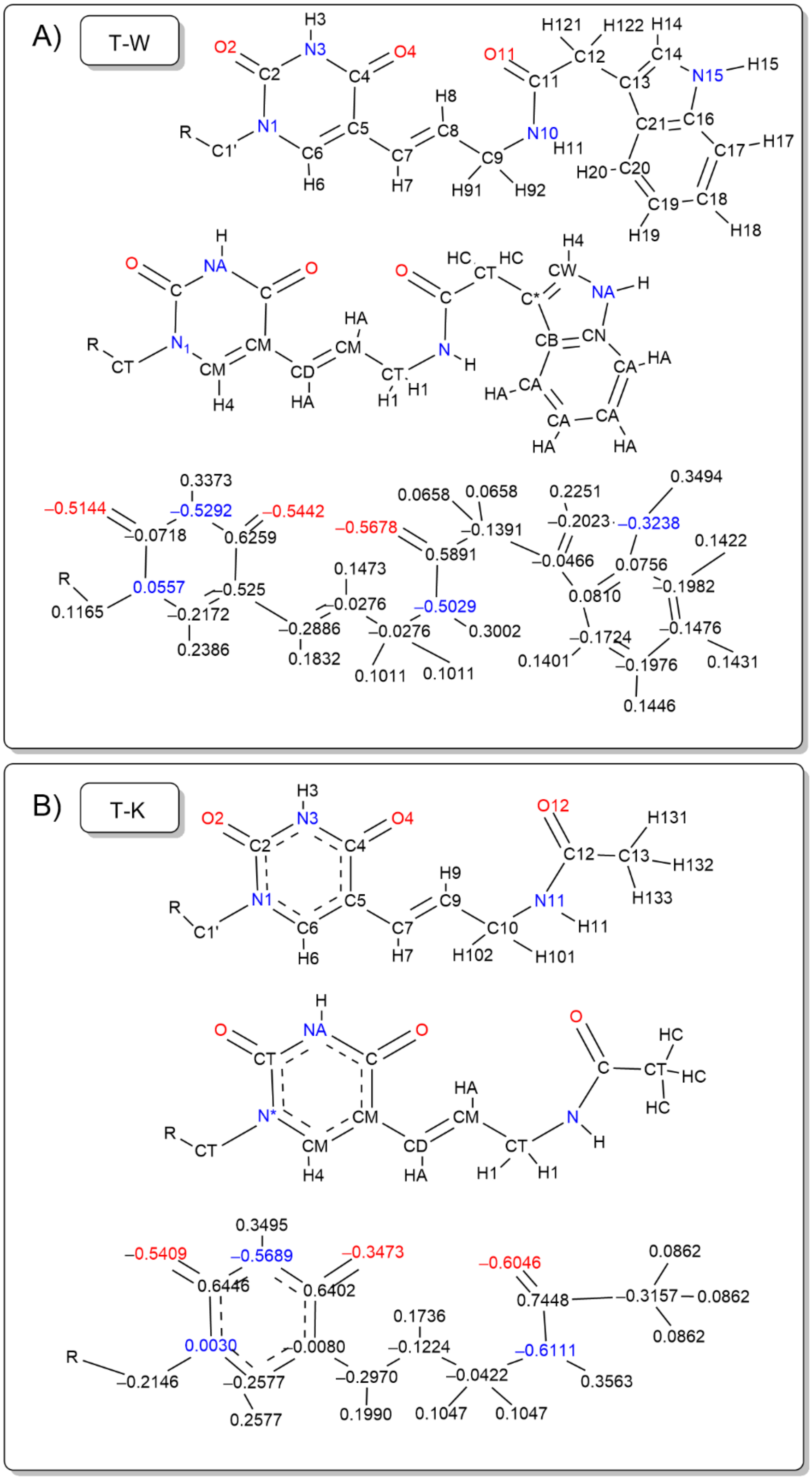

Figure S2. A) T-W and B) T-K atomic numbering (top), atom types (middle), and partial charges (bottom).

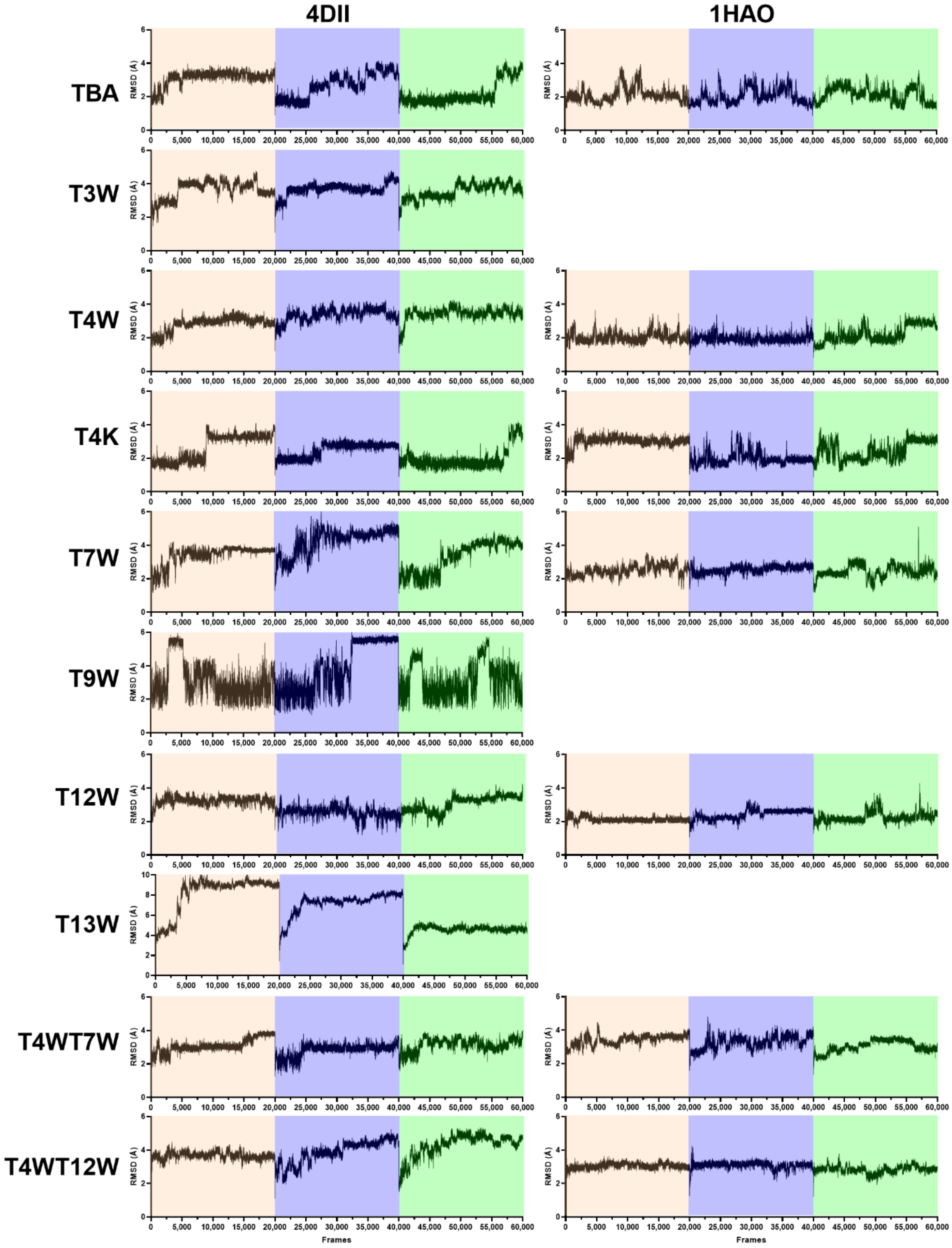

Figure S3. Heavy atom RMSDs of all canonical and modified TBA–thrombin complexes across MD simulation replicas. Replica 1 is highlighted in tan, replica 2 in lavender, and replica 3 in green.

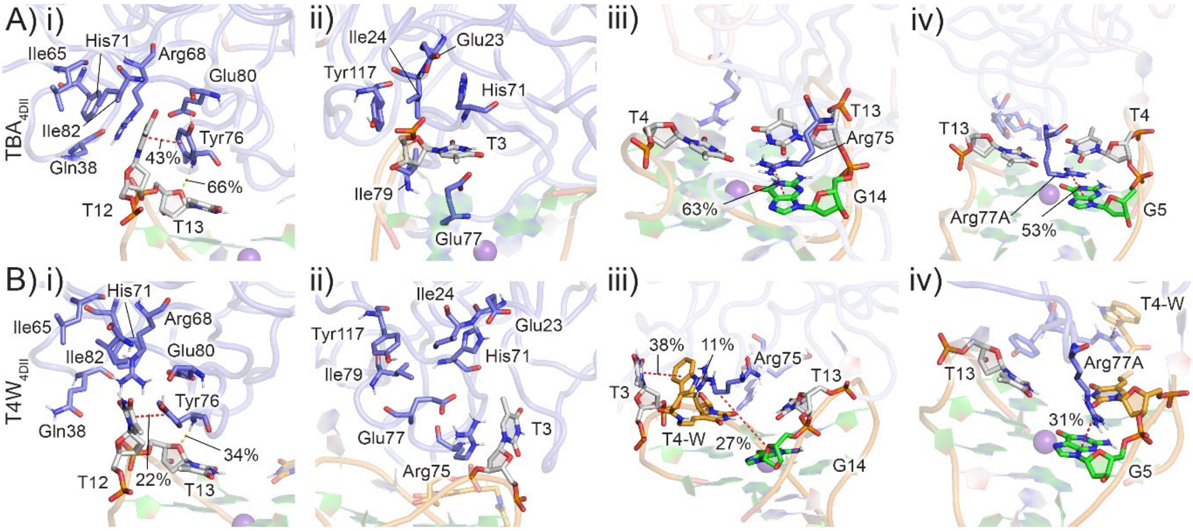

Figure S4. Key TBA–thrombin interactions observed in MD simulations initiated from PDB ID: 4DII for A) canonical TBA and B) T4W, highlighting contacts with i) Tyr76, ii) hydrophobic pocket, iii) Arg75, and iv) Arg77A. π–π interaction (red dotted lines) and hydrogen-bonding (yellow dotted lines) occupancies provided.

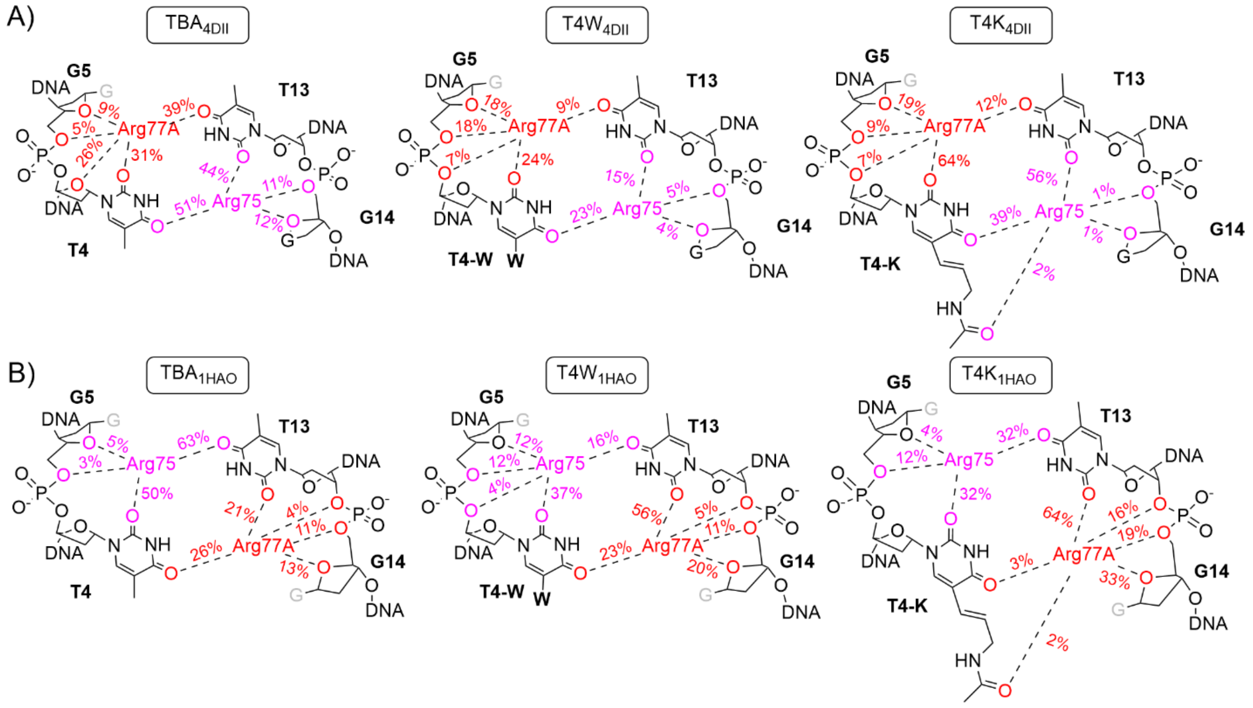

Figure S5. Occupancies of hydrogen-bonding interactions with Arg75 (magenta) and Arg77A (red) observed in MD simulations initiated from PDB ID: A) 4DII and B) 1HAO, highlighting interactions with canonical TBA (left), T4W (middle), and T4K (right).

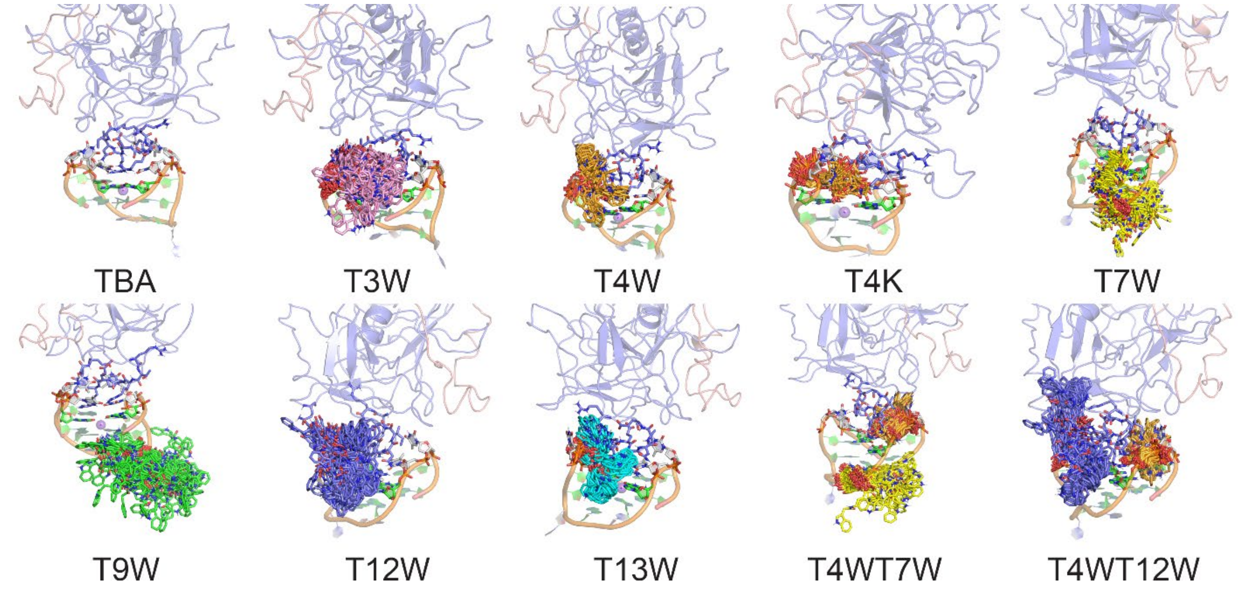

Figure S6. Overlays of T-W or T-K adducts from MD simulations initiated from PDB ID: 4DII. Overlays consist of 100 frames for each system that were evenly sampled across all replicates.

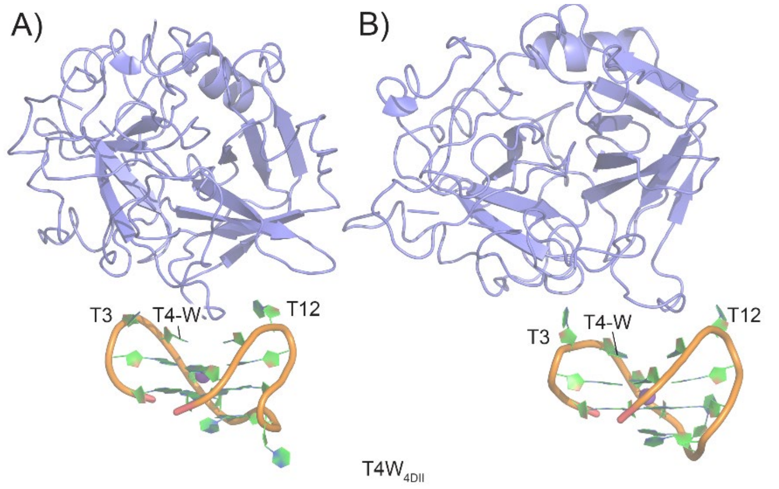

Figure S7. Representative snapshots from MD simulations of the T4W–thrombin complex initiated from PDB ID: 4DII, highlighting progression from A) an intact complex to B) unbinding of thrombin at the T12–T13 loop.

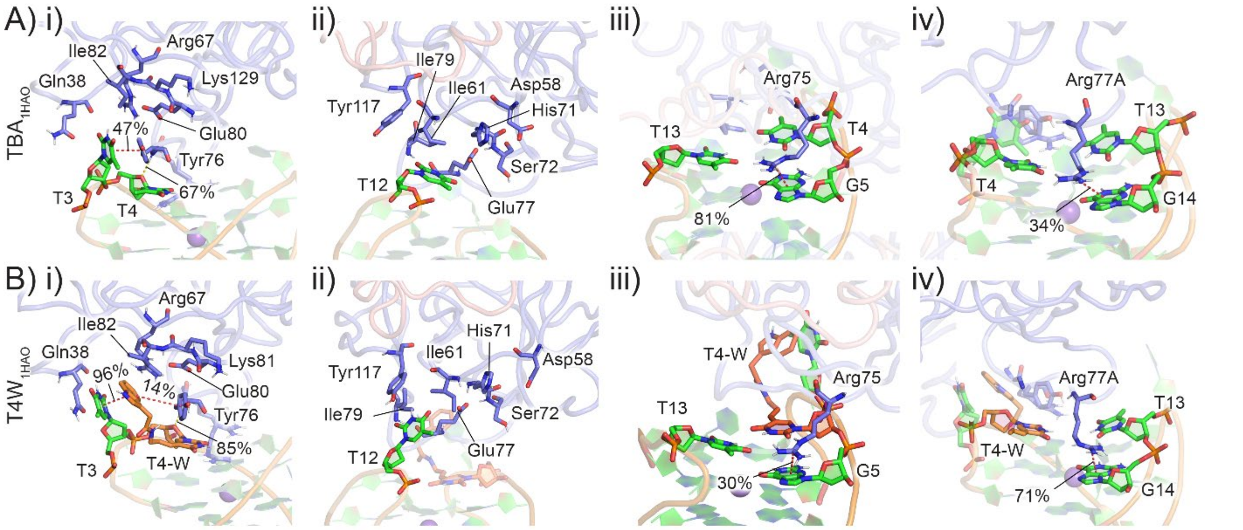

Figure S8. Key TBA–thrombin interactions observed in MD simulations initiated from PDB ID: 1HAO for A) canonical TBA and B) T4W, highlighting contacts with i) Tyr76, ii) hydrophobic pocket, iii) Arg75, and iv) Arg77A. π–π interaction (red dotted lines) and hydrogen-bonding (yellow dotted lines) occupancies provided.

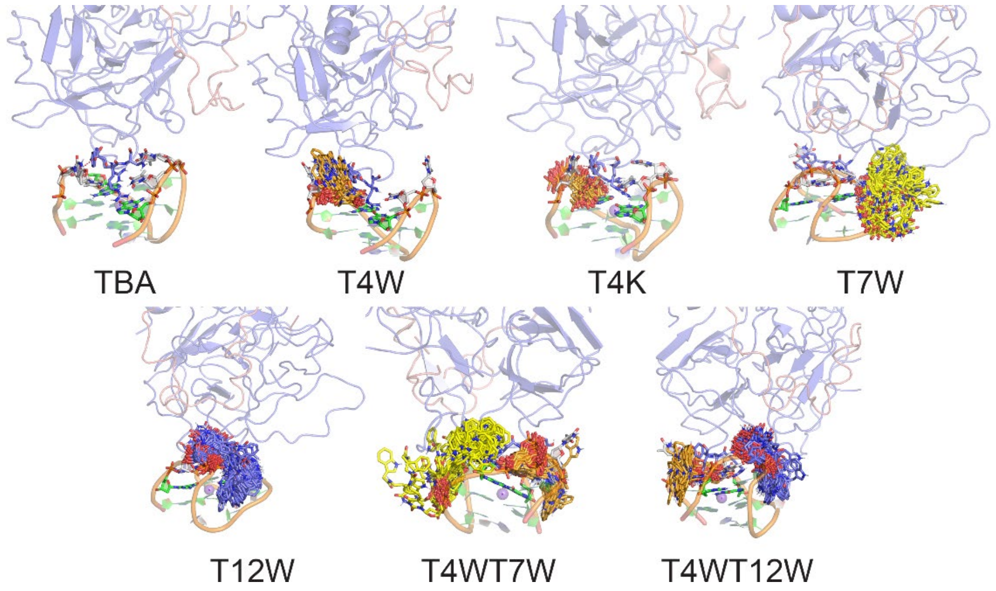

Figure S9. Overlays of T-W or T-K adducts from MD production simulation initiated from PDB ID: 1HAO. Overlays consist of 100 frames for each system that were evenly sampled across all replicates.

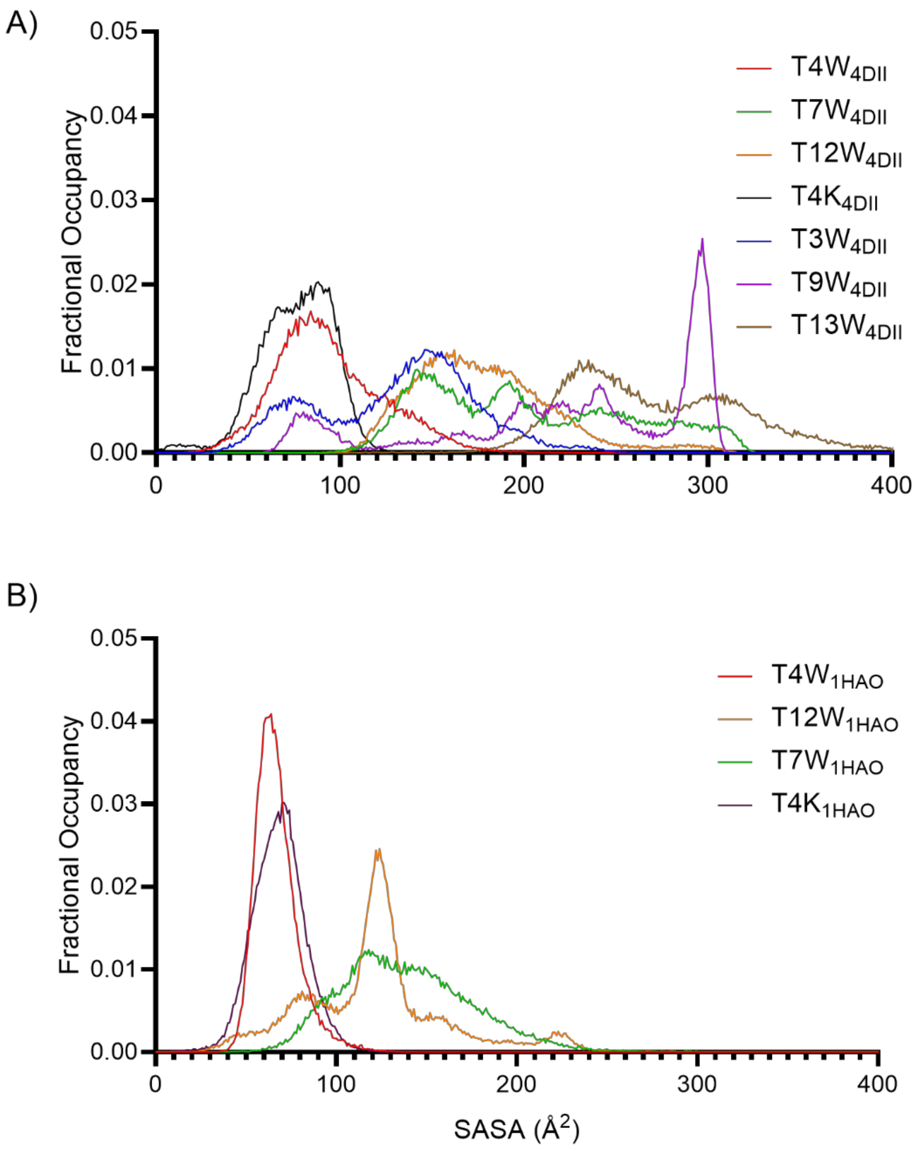

Figure S10. Fractional occupancy of solvent accessible surface area (SASA) of T-W/K at various positions in modified TBA–thrombin complexes modeled from PDB ID: A) 4DII and B) 1HAO.

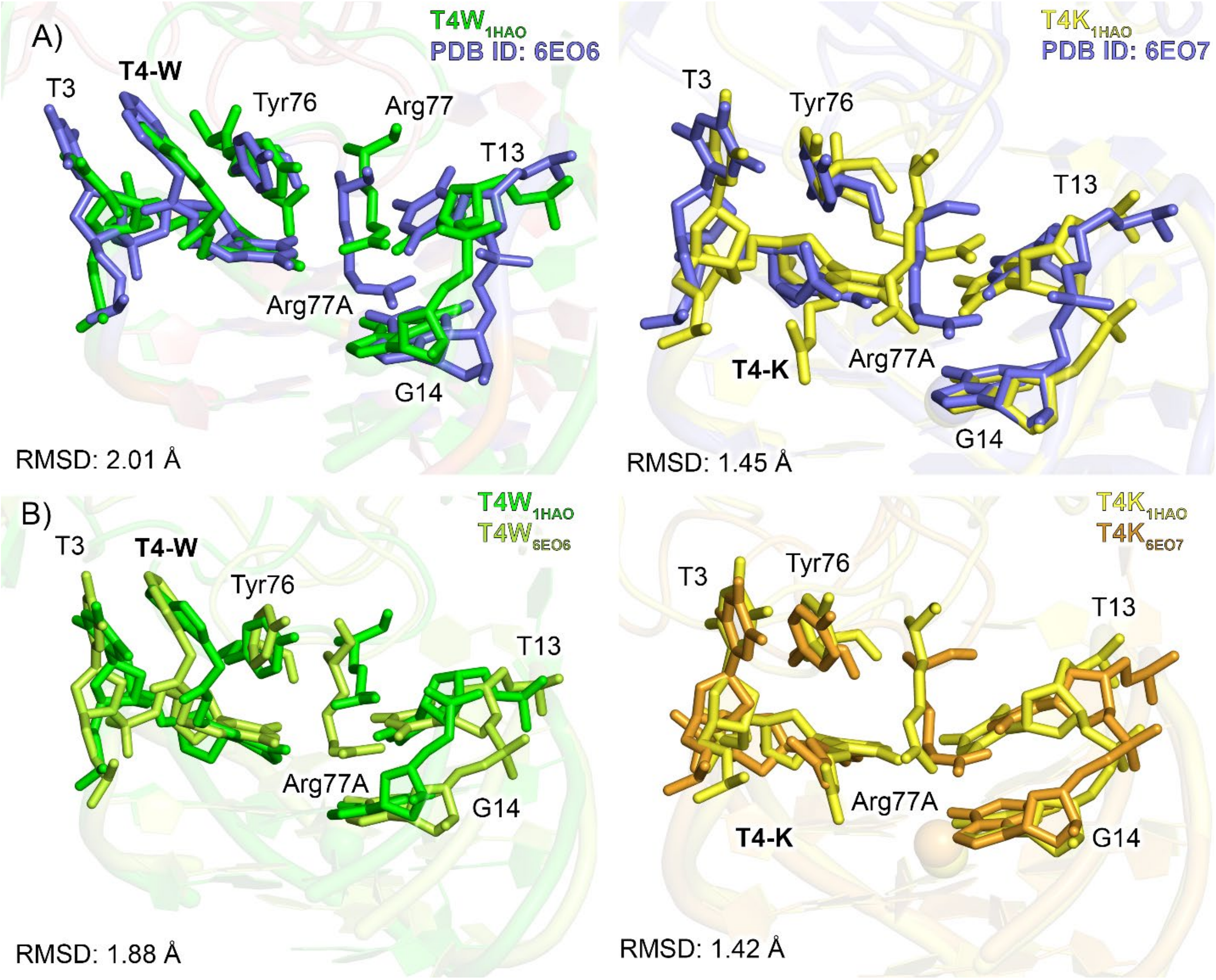

Figure S11. Overlay of the dominant MD representative structure obtained starting from the canonical TBA–thrombin complex (PDB ID: 1HAO) with A) the X-ray crystal structure or B) the MD representative structure obtained starting from the X-ray crystal structure of the modified TBA–thrombin complex for $T4W_{1HAO}$ (left) and $T4K_{1HAO}$ (right).

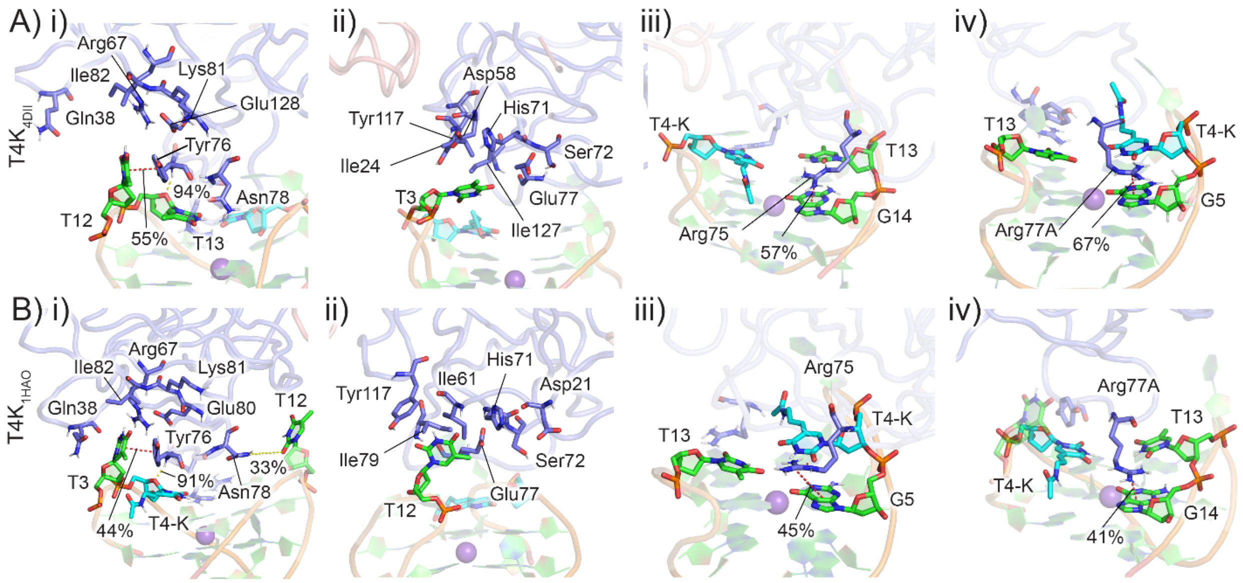

Figure S12. Key T4K–thrombin interactions observed in MD simulations initiated from PDB ID A) 4DII or B) 1HAO, highlighting contacts with i) Tyr76, ii) hydrophobic pocket, iii) Arg75, and iv) Arg77A. π–π interaction (red dotted lines) and hydrogen-bonding (yellow dotted lines) occupancies provided.

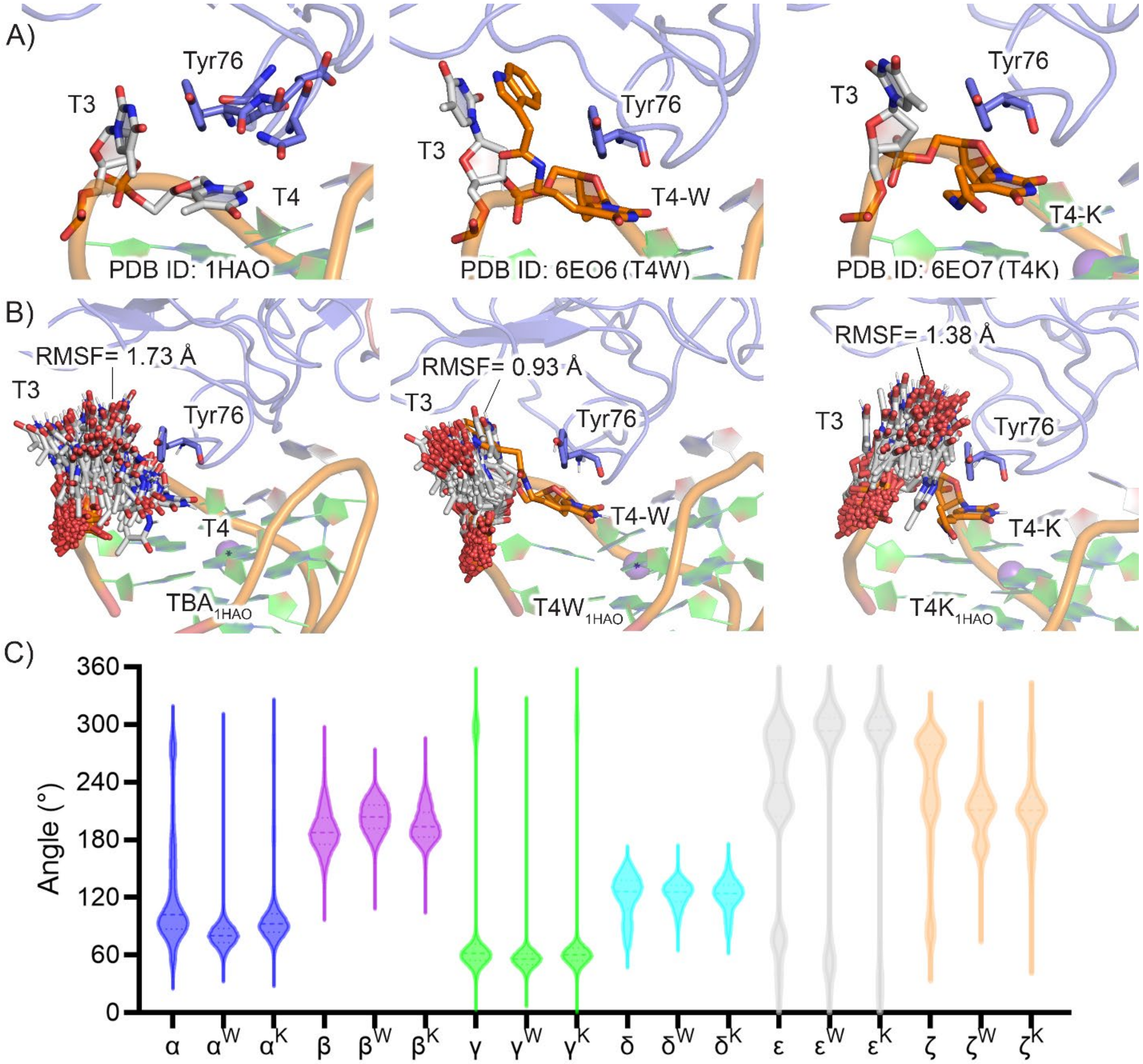

Figure S13. A) Crystal structures of thrombin bound to canonical TBA (PDB ID: 1HAO, left), T4W (PDB ID: 6EO6, center), and T4K (PDB ID: 6EO7, right), highlighting different backbone orientations at T3. B) Overlays of 100-frames for each system that were evenly sampled across all replicates. C) Violin plots of the backbone torsion angles of T3 from MD simulations of thrombin bound to canonical TBA, T4W (superscript W) or T4K (superscript K) initiated from PDB ID: 1HAO.

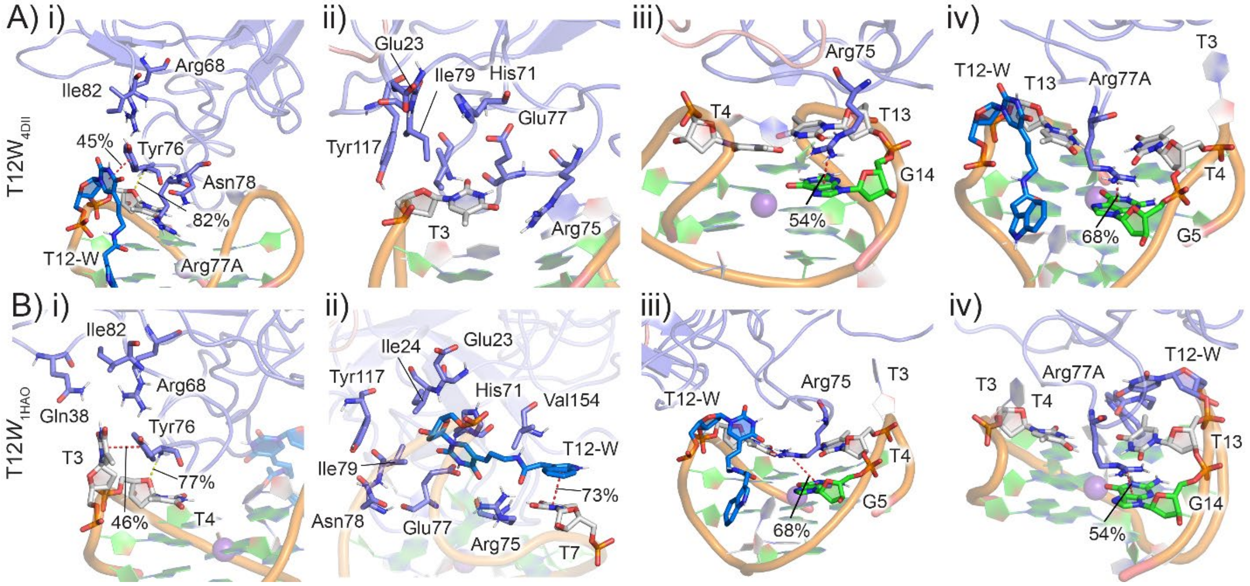

Figure S14. Key T12W–thrombin interactions observed in MD simulations initiated from PDB ID A) 4DII or B) 1HAO, highlighting contacts with i) Tyr76, ii) hydrophobic pocket, iii) Arg75, and iv) Arg77A. π–π interaction (red dotted lines) and hydrogen-bonding (yellow dotted lines) occupancies provided.

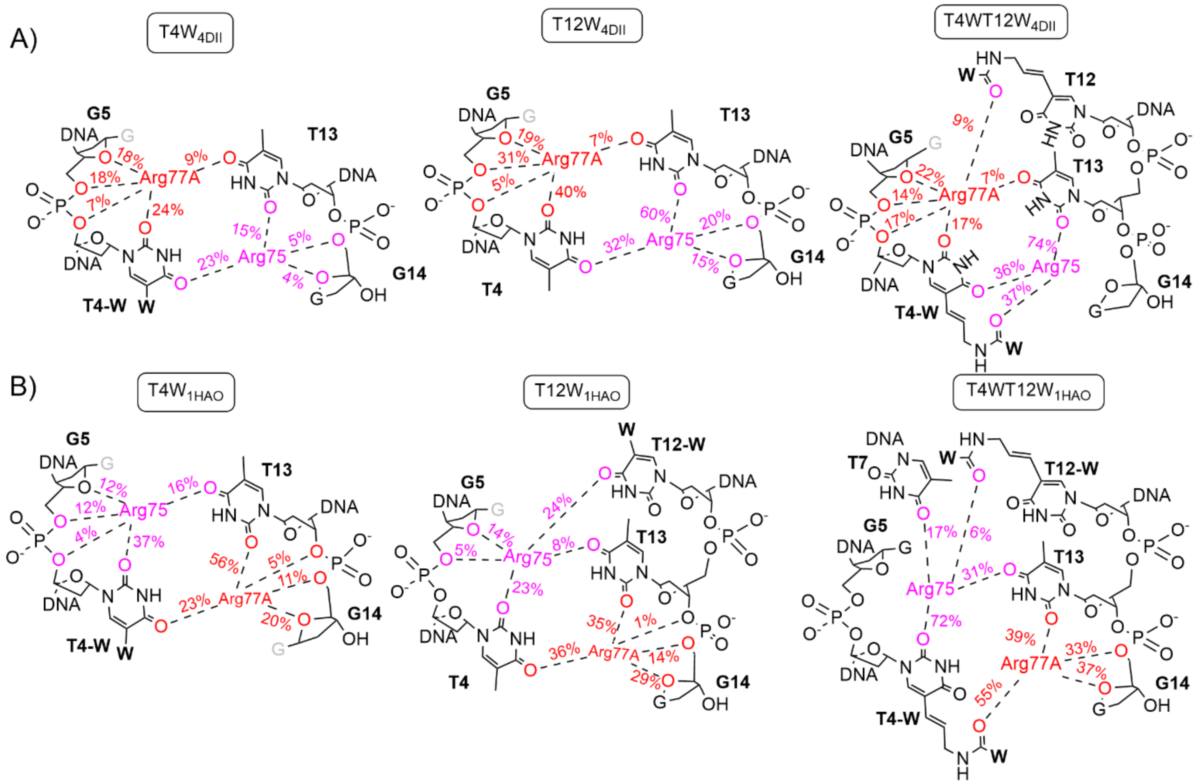

Figure S15. Occupancies of hydrogen-bonding interactions with Arg75 (magenta) and Arg77A (red) observed in MD simulations initiated from PDB ID: A) 4DII and B) 1HAO, highlighting interactions with canonical T4W (left), T12W (middle), and T4WT12W (right).

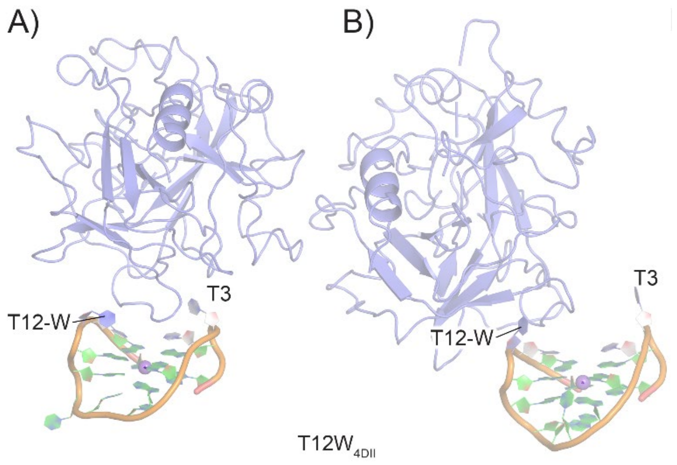

Figure S16. Representative snapshots from MD simulations of the T12W–thrombin complex initiated from PDB ID: 4DII, highlighting progression from A) an intact complex to B) unbinding of thrombin at the T3–T4 loop.

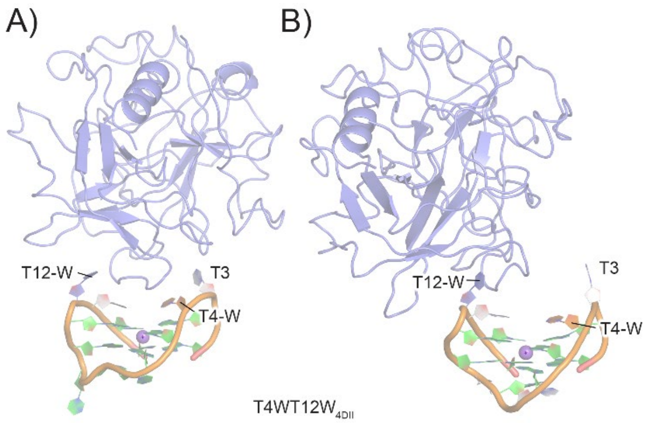

Figure S17. Representative snapshots from MD simulations of the T4WT12W–thrombin complex initiated from PDB ID: 4DII, highlighting progression from A) an intact complex to B) unbinding of thrombin at the T3–T4 loop.

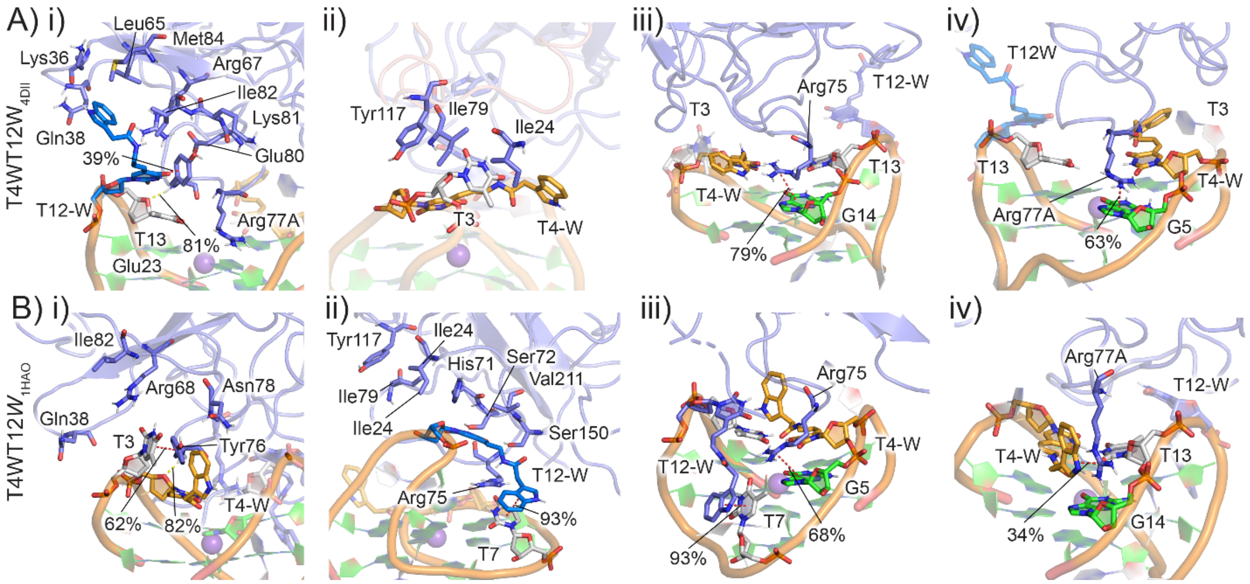

Figure S18. Key T4WT12W–thrombin interactions observed in MD simulations initiated from PDB ID: A) 4DII or B) 1HAO, highlighting contacts with i) Tyr76, ii) hydrophobic pocket, iii) Arg75, and iv) Arg77A. π–π interaction (red dotted lines) and hydrogen-bonding (yellow dotted lines) occupancies provided.

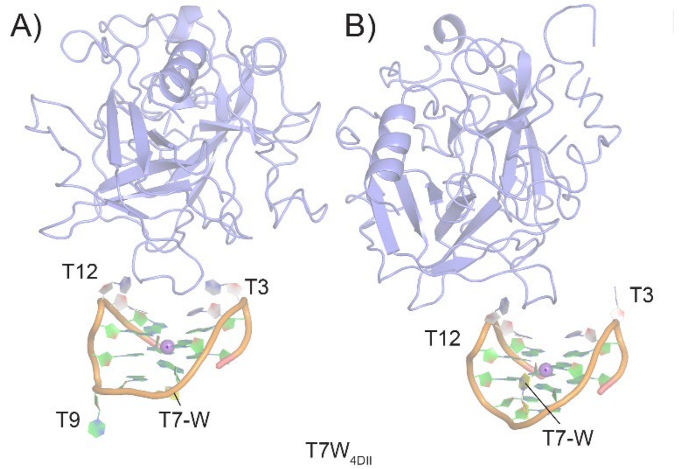

Figure S19. Representative snapshots from MD simulations of the T7W–thrombin complex initiated from PDB ID: 4DII, highlighting progression from A) an intact complex to B) unbinding of thrombin at the T3–T4 loop.

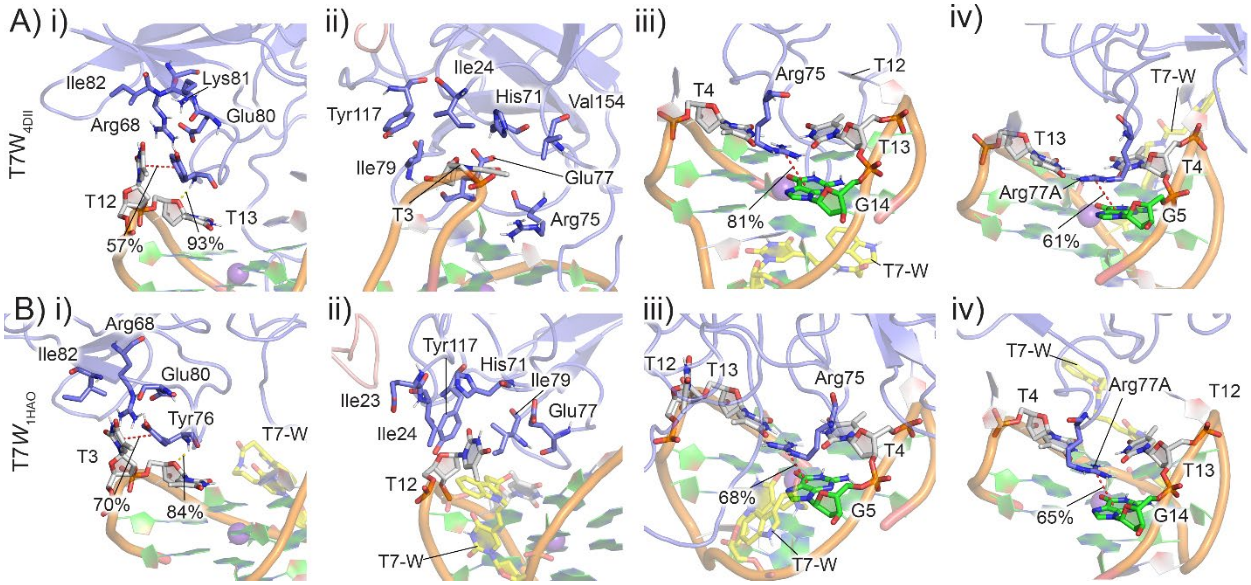

Figure S20. Key T7W–thrombin interactions observed in MD simulations initiated from PDB ID: A) 4DII or B) 1HAO, highlighting contacts with i) Tyr76, ii) Hydrophobic pocket, iii) Arg75, and iv) Arg77A. π–π interaction (red dotted lines) and hydrogen-bonding (yellow dotted lines) occupancies provided.

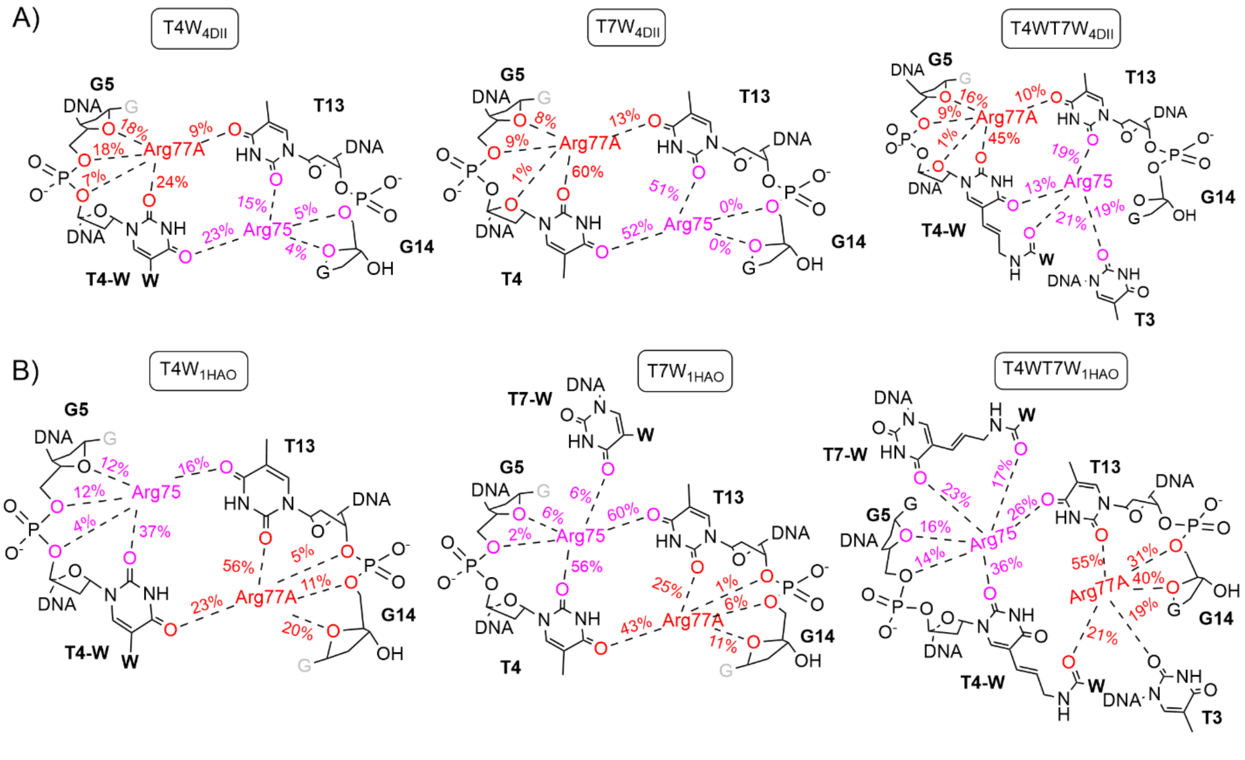

Figure S21. Occupancies of hydrogen-bonding interactions with Arg75 (magenta) and Arg77A (red) observed in MD simulations initiated from PDB ID: A) 4DII and B) 1HAO, highlighting interactions with canonical T4W (left), T7W (middle), and T4WT7W (right).

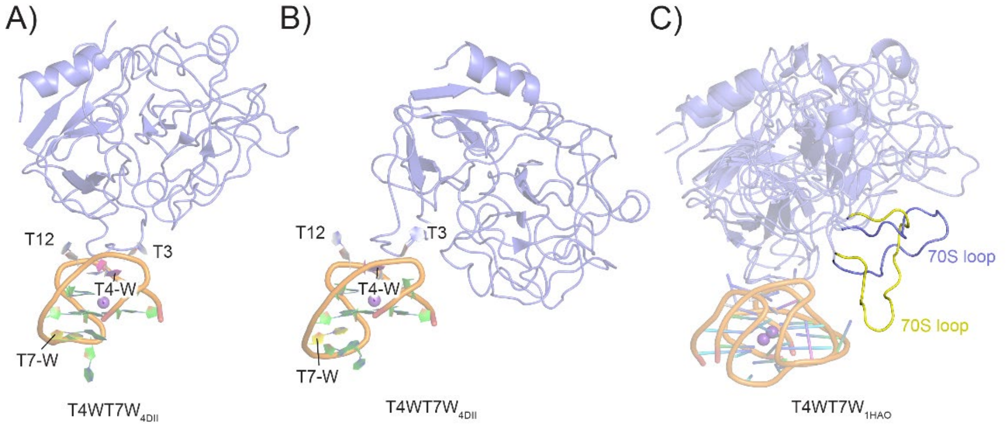

Figure S22. Representative snapshots from MD simulations of the T4WT7W–thrombin complex initiated from PDB ID: 4DII, highlighting progression from A) an intact complex to B) unbinding of thrombin at the T12–T13 loop. C) The movement of the 70S loop from TBA_{1HAO} (blue) to $T4WT7W_{1HAO}$ (yellow).

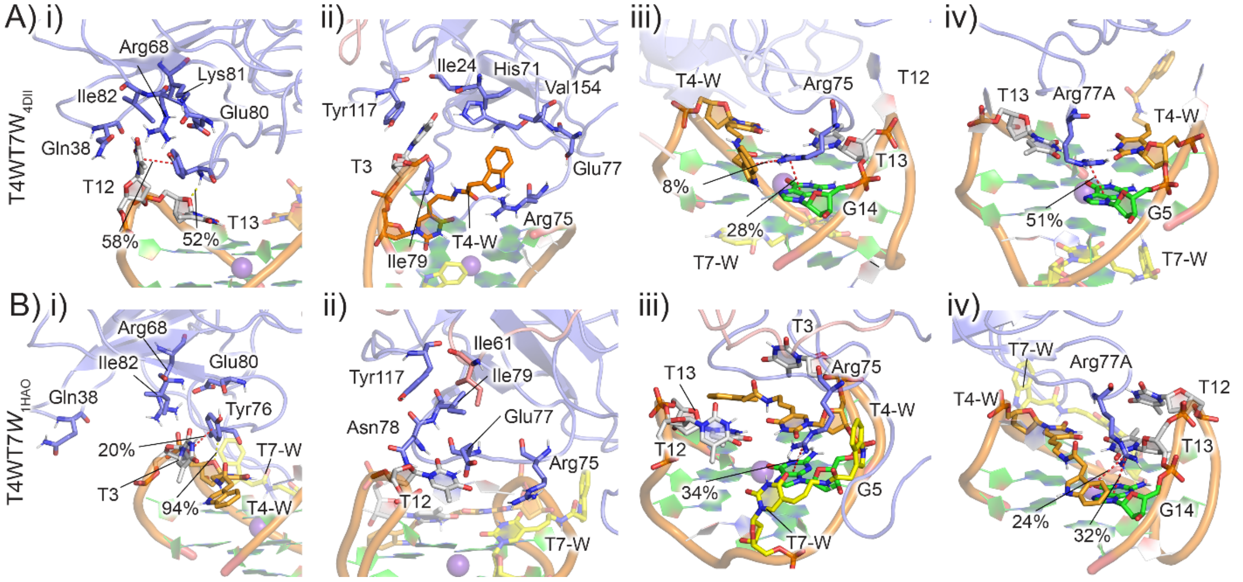

Figure S23. Key T4WT7W–thrombin interactions observed in MD simulations initiated from PDB ID: A) 4DII or B) 1HAO, highlighting contacts with i) Tyr76, ii) hydrophobic pocket, iii) Arg75, and iv) Arg77A. π–π interaction (red dotted lines) and hydrogen-bonding (yellow dotted lines) occupancies provided.

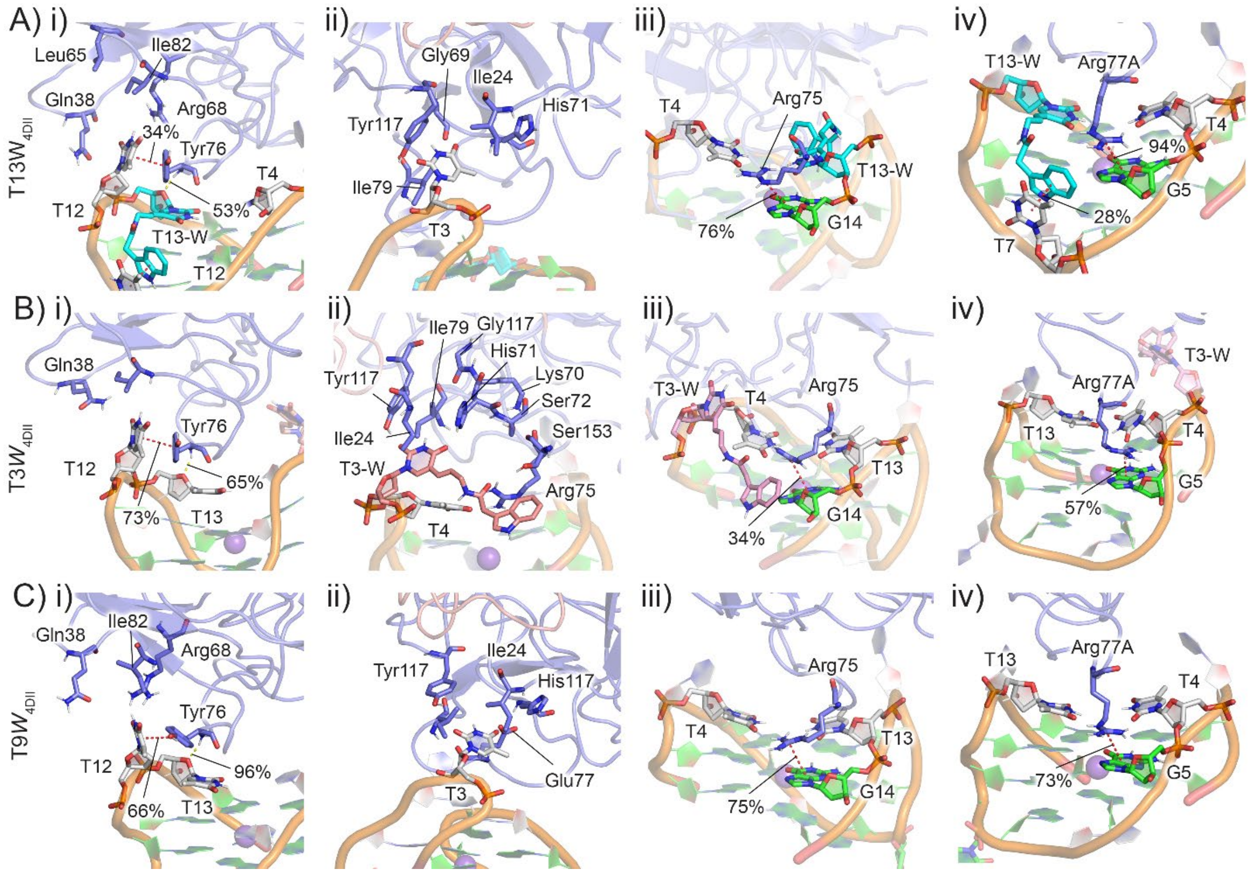

Figure S24. Key T4WT7W–thrombin interactions observed in MD simulations initiated from PDB ID: 4DII, A) T13W, B) T3W, and C) T9W, highlighting contacts with i) T12 and Tyr76, ii) T3 and a hydrophobic pocket, iii) Arg75, and iv) Arg77A. π–π interaction (red dotted lines) and hydrogen-bonding (yellow dotted lines) occupancies provided.

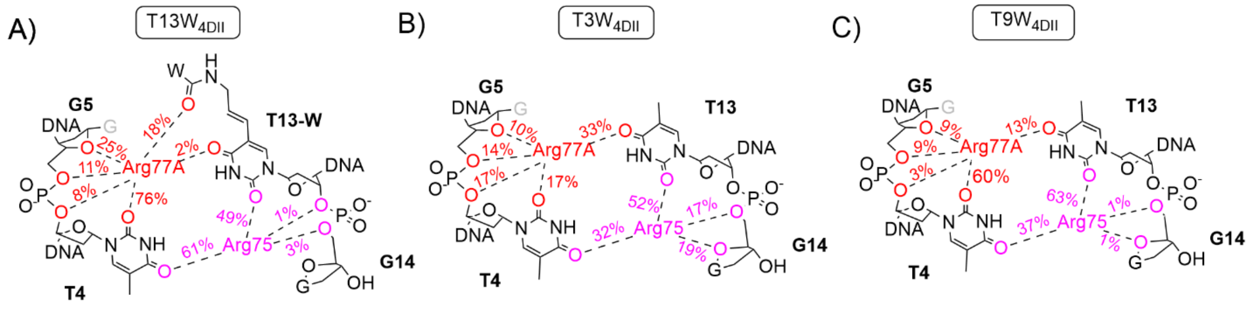

Figure S25. Occupancies of hydrogen-bonding interactions with of Arg75 (magenta) and Arg77A (red) observed in MD simulations initiated from PDB ID: 4DII, highlighting interactions with A) T13W, B) T3W, and C) T9W.

Table S1. Unresolved thrombin residues in X-ray crystal structures of TBA–thrombin complexes that were added during model building.[a]

Crystal Structure	Missing Residues
**4DII**	T-5, F-4, G-3, S-2, G-1, E0, D14L, G14M, R14N, G148, K149, G150, E247
**1HAO**	T-5, F-4, G-3, S-2, G-1, E0, D14L, G14M, R14N, K149
**6EO6**	T328, F329, G330, S331, G332, G362, R363,
**6EO7**	T328, F329, G330, S331, G362, R363,

[a] Residue numbering reflects the corresponding crystal structure numbering. The first resolved residue for PDB ID: 4DII and 1HAO is reside '1', with unresolved residues prior to '1' labeled as negative numbers. Residues were added to models using Pymol 2.5

Table S2. Summary of aptamers and thrombin binding orientations considered in the present work.

System Name	TBA Sequence[a]	Binding Orientation[b] 4DII	1HAO
**TBA**	5'-GG-TT-GG-TGT-GG-TT-GG-3'	✓	✓
**T3W**	5'-GG-**W**T-GG-TGT-GG-TT-GG-3'	✓	
**T4W**	5'-GG-T**W**-GG-TGT-GG-TT-GG-3'	✓	✓
**T4K**	5'-GG-T**K**-GG-TGT-GG-TT-GG-3'	✓	✓
**T7W**	5'-GG-TT-GG-**W**GT-GG-TT-GG-3'	✓	✓
**T9W**	5'-GG-TT-GG-TG**W**-GG-TT-GG-3'	✓	
**T12W**	5'-GG-TT-GG-TGT-GG-**W**T-GG-3'	✓	✓
**T13W**	5'-GG-TT-GG-TGT-GG-T**W**-GG-3'	✓	
**T4WT7W**	5'-GG-T**W**-GG-**W**GT-GG-TT-GG-3'	✓	✓
**T4WT12W**	5'-GG-T**W**-GG-TGT-GG-**W**T-GG-3'	✓	✓

[a] Numbering of residues starts on the left. [b] Binding orientation found in the X-ray crystal structure with PDB ID: 4DII and 1HAO.

Table S3. Number of ions and water molecules added to each aptamer–thrombin model.[a]

	4DII			1HAO		
**System[b]**	**Water**	**Na^+**	**Cl^-**	**Water**	**Na^+**	**Cl^-**
**TBA**	11,814	33	22	10,779	30	19
**T3W**	11,823	35	24	—	—	—
**T4W**	11,881	34	23	10,837	32	21
**T4K**	11,829	33	22	10,820	32	21
**T7W**	15,850	45	34	10,429	31	18
**T9W**	17,078	48	37	—	—	—
**T12W**	11,679	34	23	10,766	32	21
**T13W**	11,881	34	23	—	—	—
**T4WT7W**	16,545	47	36	9,280	28	17
**T4WT12W**	11,712	34	23	10,644	31	20

[a] Number of ions determined using the SLTCAP calculator. [b] System represents the aptamer bound to thrombin.

Table S4. Percent occupancy, standard deviation, and standard error for key π–π stacking and hydrogen-bonding interactions in canonical and singly modified aptamers with enhanced binding affinity.[a]

System	T···Tyr76 Stack			Arg77A···G Stack			Tyr76···T Hbond			Arg77A Hbond			Arg75 Hbond		
	% Occ.	Std.Dev.	Std.Err.	% Occ.	Std.Dev.	Std.Err.	% Occ.	Std.Dev.	Std.Err.	% Occ.	Std.Dev.	Std.Err.	% Occ..	Std.Dev.	Std.Err.
**TBA_{4DII}**	43%	26%	1%	55%	40%	2%	70%	39%	2%	27%	41%	2%	8%	24%	1%
**TBA_{1HAO}**	55%	33%	1%	38%	33%	1%	67%	40%	2%	51%	47%	2%	27%	38%	2%
**$T4W_{4DII}$**	19%	29%	1%	55%	40%	2%	32%	41%	2%	23%	40%	2%	16%	26%	1%
**$T4W_{1HAO}$**	34%	17%	1%	72%	31%	1%	85%	13%	1%	23%	40%	2%	60%	45%	2%
**$T4K_{4DII}$**	55%	20%	1%	72%	29%	1%	94%	8%	0%	2%	12%	0%	63%	44%	2%
**$T4K_{1HAO}$**	32%	25%	1%	41%	46%	2%	63%	40%	2%	39%	44%	2%	12%	28%	1%
**$T12W_{1HAO}$**	46%	31%	1%	62%	41%	2%	78%	23%	1%	8%	21%	1%	10%	28%	1%
**$T7W_{1HAO}$**	70%	24%	1%	65%	34%	1%	84%	27%	1%	42%	46%	2%	39%	47%	2%

[a] Statistics were calculated using block averaging according to blocks of 100 frames (5 ns), resulting in 600 blocks per interaction across all replicates.

Table S5. Results from one-way ANOVA for key π–π stacking and hydrogen-bonding interactions grouped across canonical and singly modified aptamers with enhanced binding affinity.[a]

		Arg77A···G Stack	T···Tyr76 Stack	Tyr76···T Hbond	Arg77A Hbond	Arg75 Hbond
**Treatment between columns**	**Sum of Squares**	68.62	110.7	153.2	119.8	206.9
	**Degrees of Freedom**	7	7	7	7	7
	**Mean Square**	9.802	15.81	21.89	17.12	29.55
	**F Statistic**	71.3	242.4	220.5	117.4	227.4
	**P value**	1.8577×10^{-98}	9.0749×10^{-310}	7.5617×10^{-285}	1.5938×10^{-159}	8.7192×10^{-293}
**Residual within columns**	**Sum of Squares**	658.8	313	475.8	699.1	622.7
	**Degrees of Freedom**	4792	4800	4792	4792	4792
	**Mean Square**	0.1375	0.06522	0.09928	0.1459	0.1299

[a] Prism software was used to perform one-way ANOVA across the block averaged datasets for each interaction type.

Table S6. Adjusted P values from Tukey's tests for key aptamer–thrombin π–π stacking and hydrogen-bonding interactions.[a,b]

**Tukey's Test Pairings**		**T···Tyr76 Stack**	**Arg77A···G Stack**	**Tyr76···T Hbond**	**Arg77A Hbond**	**Arg75 Hbond**
**TBA_{4DII}**	**TBA_{1HAO}**	2.704×10^{-8}	0.1646	0.9062	2.704×10^{-8}	2.704×10^{-8}
**TBA_{4DII}**	**$T4W_{4DII}$**	>0.9999	2.610×10^{-8}	2.704×10^{-8}	2.704×10^{-8}	2.704×10^{-8}
**TBA_{4DII}**	**$T4W_{1HAO}$**	2.704×10^{-8}	8.229×10^{-8}	2.704×10^{-8}	2.704×10^{-8}	2.704×10^{-8}
**TBA_{4DII}**	**$T4K_{4DII}$**	2.704×10^{-8}	2.610×10^{-8}	2.704×10^{-8}	1.641×10^{-6}	2.704×10^{-8}
**TBA_{4DII}**	**$T4K_{1HAO}$**	2.877×10^{-8}	2.610×10^{-8}	0.0030	2.704×10^{-8}	1.457×10^{-7}
**TBA_{4DII}**	**$T7W_{1HAO}$**	1.195×10^{-4}	2.610×10^{-8}	2.704×10^{-8}	0.0016	2.704×10^{-8}
**TBA_{4DII}**	**$T12W_{1HAO}$**	0.0306	0.2218	2.44×10^{-4}	2.704×10^{-8}	2.704×10^{-8}
**TBA_{1HAO}**	**$T4W_{4DII}$**	2.704×10^{-8}	2.610×10^{-8}	2.704×10^{-8}	0.8245	2.704×10^{-8}
**TBA_{1HAO}**	**$T4W_{1HAO}$**	2.704×10^{-8}	2.610×10^{-8}	2.704×10^{-8}	0.7451	2.704×10^{-8}
**TBA_{1HAO}**	**$T4K_{4DII}$**	2.704×10^{-8}	3.671×10^{-7}	2.704×10^{-8}	2.568×10^{-7}	2.704×10^{-8}
**TBA_{1HAO}**	**$T4K_{1HAO}$**	0.9107	2.610×10^{-8}	0.1679	2.704×10^{-8}	2.704×10^{-8}
**TBA_{1HAO}**	**$T7W_{1HAO}$**	2.704×10^{-8}	2.610×10^{-8}	2.704×10^{-8}	2.706×10^{-8}	0.8210
**TBA_{1HAO}**	**$T12W_{1HAO}$**	2.704×10^{-8}	>0.9999	3.367×10^{-7}	2.704×10^{-8}	2.704×10^{-8}
**$T4W_{4DII}$**	**$T4W_{1HAO}$**	2.704×10^{-8}	2.610×10^{-8}	2.704×10^{-8}	>0.9999	0.0374
**$T4W_{4DII}$**	**$T4K_{4DII}$**	2.704×10^{-8}	2.610×10^{-8}	2.704×10^{-8}	2.705×10^{-8}	0.9970
**$T4W_{4DII}$**	**$T4K_{1HAO}$**	2.877×10^{-8}	2.610×10^{-8}	2.704×10^{-8}	2.704×10^{-8}	2.704×10^{-8}
**$T4W_{4DII}$**	**$T7W_{1HAO}$**	1.195×10^{-4}	2.610×10^{-8}	2.704×10^{-8}	2.704×10^{-8}	2.704×10^{-8}
**$T4W_{4DII}$**	**$T12W_{1HAO}$**	0.0306	2.610×10^{-8}	2.704×10^{-8}	2.707×10^{-8}	0.9970
**$T4W_{1HAO}$**	**$T4K_{4DII}$**	>0.9999	2.610×10^{-8}	1.552×10^{-5}	2.704×10^{-8}	0.5979
**$T4W_{1HAO}$**	**$T4K_{1HAO}$**	2.704×10^{-8}	0.6849	2.704×10^{-8}	2.704×10^{-8}	1.302×10^{-5}
**$T4W_{1HAO}$**	**$T7W_{1HAO}$**	0.0207	2.610×10^{-8}	0.9959	2.704×10^{-8}	2.704×10^{-8}
**$T4W_{1HAO}$**	**$T12W_{1HAO}$**	6.680×10^{-5}	2.610×10^{-8}	9.074×10^{-4}	2.711×10^{-8}	0.0033
**$T4K_{4DII}$**	**$T4K_{1HAO}$**	2.704×10^{-8}	2.610×10^{-8}	2.704×10^{-8}	2.704×10^{-8}	2.723×10^{-8}
**$T4K_{4DII}$**	**$T7W_{1HAO}$**	0.0230	2.610×10^{-8}	2.966×10^{-7}	0.8436	2.704×10^{-8}
**$T4K_{4DII}$**	**$T12W_{1HAO}$**	7.808×10^{-5}	1.793×10^{-07}	2.704×10^{-8}	2.704×10^{-8}	0.4830
**$T4K_{1HAO}$**	**$T7W_{1HAO}$**	2.704×10^{-8}	2.610×10^{-8}	2.704×10^{-8}	2.704×10^{-8}	2.704×10^{-8}
**$T4K_{1HAO}$**	**$T12W_{1HAO}$**	2.704×10^{-8}	2.610×10^{-8}	2.704×10^{-8}	0.2520	2.704×10^{-8}
**$T7W_{1HAO}$**	**$T12W_{1HAO}$**	0.8568	2.610×10^{-8}	0.0152	2.704×10^{-8}	2.704×10^{-8}

[a] Tukey's test was performed due to a relatively high degree of freedom from one-way ANOVA (Table S5). No dataset is compared to itself and order permutations were not considered. [b] Dataset pairs with P values < 0.05 were determined to be significantly distinct and discussed as such in the main text.

Table S7. RMSF values averaged across simulation replicas for each nucleotide in the aptamer bound to thrombin in the orientation found in the X-ray crystal structures with PDB ID: 4DII and 1HAO.[a]

Binding Pose	Residues	TBA	T3W	T4W	T4K	T7W	T9W	T12W	T13W	T4WT7W	T4WT12W
4DII	G1	0.836	0.896	0.791	0.910	1.307	1.182	0.864	0.864	0.800	0.934
	G2	0.815	0.910	0.762	0.898	1.114	1.207	0.836	0.836	0.880	0.840
	T3	2.285	2.352	1.437	2.002	2.450	2.458	2.104	2.104	2.340	1.682
	T4	1.145	1.003	1.884	1.331	1.461	1.547	1.262	1.262	2.007	1.582
	G5	0.888	0.935	0.881	0.949	1.333	1.072	1.037	1.037	0.878	1.228
	G6	0.942	1.029	0.909	0.927	1.522	1.107	1.130	1.130	0.880	1.506
	T7	3.118	3.223	3.444	2.852	5.079	1.743	2.688	2.688	2.329	3.758
	G8	2.928	2.417	2.482	2.437	3.015	2.491	2.989	2.989	2.548	2.381
	T9	3.408	2.825	2.647	3.740	3.373	4.845	3.840	3.840	2.972	2.500
	G10	0.982	1.023	0.975	1.0779	1.215	1.309	1.159	1.159	0.993	1.071
	G11	0.797	0.814	0.780	0.849	1.046	1.072	1.087	1.087	0.820	1.007
	T12	1.921	1.634	1.564	1.350	2.040	1.583	3.250	3.250	1.459	3.888
	T13	1.151	1.043	0.992	0.950	1.222	0.988	1.102	1.102	0.971	1.291
	G14	0.921	0.991	0.834	0.944	1.047	1.006	0.887	0.887	0.915	0.896
	G15	1.053	1.276	0.995	1.347	1.388	1.318	1.064	1.064	1.034	1.063
1HAO	G1	0.631	—	0.607	0.724	0.582	—	0.556	—	0.735	0.595
	G2	0.647	—	0.602	0.681	0.690	—	0.621	—	0.812	0.603
	T3	1.732	—	0.931	1.380	1.607	—	1.681	—	1.888	1.475
	T4	0.954	—	1.003	0.979	0.964	—	1.012	—	1.874	1.502
	G5	0.786	—	0.766	0.813	0.758	—	0.679	—	0.893	0.669
	G6	0.882	—	0.853	0.936	0.740	—	0.719	—	0.919	0.659
	T7	3.058	—	2.635	3.636	2.795	—	2.124	—	2.764	1.769
	G8	2.271	—	1.893	2.386	1.504	—	1.942	—	1.504	2.010
	T9	2.133	—	1.513	2.224	1.471	—	1.511	—	1.596	1.601
	G10	0.811	—	0.686	0.902	0.775	—	0.727	—	0.861	0.758
	G11	0.759	—	0.605	0.820	0.798	—	0.656	—	0.841	0.698
	T12	2.121	—	1.461	1.997	2.265	—	2.201	—	2.867	2.042
	T13	1.094	—	0.967	1.214	1.100	—	1.338	—	1.152	1.176
	G14	0.789	—	0.795	0.851	0.782	—	0.796	—	0.755	0.776
	G15	0.808	—	0.764	0.958	0.775	—	0.730	—	0.821	0.728

[a] RMSF values calculated over all heavy atoms in the residue of interest.

References

1. Russo Krauss, I., Merlino, A., Randazzo, A., Novellino, E., Mazzarella, L. and Sica, F. (2012) High-resolution structures of two complexes between thrombin and thrombin-binding aptamer shed light on the role of cations in the aptamer inhibitory activity. Nucleic Acids Res., 40, 8119-8128.
2. Padmanabhan, K. and Tulinsky, A. (1996) An Ambiguous Structure of a DNA 15-mer Thrombin Complex. Acta Crystallogr. Sect. D. Biol. Crystallogr., 52, 272-282.
3. DeLano, W.L. (2002) Pymol: An open-source molecular graphics tool. CCP4 Newsl. Protein Crystallogr., 40, 82-92.
4. Ponder, J.W. and Case, D.A. (2003) Force fields for protein simulations. Adv. Protein Chem., 66, 27-85.
5. Froimowitz, M. (1993) HyperChem: A software package for computational chemistry and molecular modeling. BioTechniques, 14, 1010-1013.
6. Frisch, M.J., Trucks, G.W., Schlegel, H.B., Scuseria, G.E., Robb, M.A., Cheeseman, J.R., Scalmani, G., Barone, V., Petersson, G.A., Nakatsuji, H., Li, X., Caricato, M., Marenich, A.V., Bloino, J., Janesko, B.G., Gomperts, R., Mennucci, B., Hratchian, H.P., Ortiz, J.V., Izmaylov, A.F., Sonnenberg, J.L., Williams, Ding, F., Lipparini, F., Egidi, F., Goings, J., Peng, B., Petrone, A., Henderson, T., Ranasinghe, D., Zakrzewski, V.G., Gao, J., Rega, N., Zheng, G., Liang, W., Hada, M., Ehara, M., Toyota, K., Fukuda, R., Hasegawa, J., Ishida, M., Nakajima, T., Honda, Y., Kitao, O., Nakai, H., Vreven, T., Throssell, K., Montgomery Jr., J.A., Peralta, J.E., Ogliaro, F., Bearpark, M.J., Heyd, J.J., Brothers, E.N., Kudin, K.N., Staroverov, V.N., Keith, T.A., Kobayashi, R., Normand, J., Raghavachari, K., Rendell, A.P., Burant, J.C., Iyengar, S.S., Tomasi, J., Cossi, M., Millam, J.M., Klene, M., Adamo, C., Cammi, R., Ochterski, J.W., Martin, R.L., Morokuma, K., Farkas, O., Foresman, J.B. and Fox, D.J. (2016) Gaussian 16 Rev. C.01.
7. Case, D.A., Aktulga, H.M., Belfon, K., Cerutti, D.S., Cisneros, G.A., Cruzeiro, V.W.D., Forouzesh, N., Giese, T.J., Götz, A.W., Gohlke, H., Izadi, S., Kasavajhala, K., Kaymak, M.C., King, E., Kurtzman, T., Lee, T.-S., Li, P., Liu, J., Luchko, T., Luo, R., Manathunga, M., Machado, M.R., Nguyen, H.M., O'Hearn, K.A., Onufriev, A.V., Pan, F., Pantano, S., Qi, R., Rahnamoun, A., Risheh, A., Schott-Verdugo, S., Shajan, A., Swails, J., Wang, J., Wei, H., Wu, X., Wu, Y., Zhang, S., Zhao, S., Zhu, Q., Cheatham, T.E., III, Roe, D.R., Roitberg, A., Simmerling, C., York, D.M., Nagan, M.C. and Merz, K.M., Jr. (2023) AmberTools. J. Chem. Inf. Model., 63, 6183-6191.
8. Schmit, J.D., Kariyawasam, N.L., Needham, V. and Smith, P.E. (2018) SLTCAP: A simple method for calculating the number of ions needed for MD simulation. J. Chem. Theory Comput., 14, 1823-1827.
9. Zgarbová, M., Šponer, J., Otyepka, M., Cheatham, T.E., Galindo-Murillo, R. and Jurečka, P. (2015) Refinement of the sugar–phosphate backbone torsion beta for AMBER force fields improves the description of Z- and B-DNA. J. Chem. Theory Comput., 11, 5723-5736.
10. Maier, J.A., Martinez, C., Kasavajhala, K., Wickstrom, L., Hauser, K.E. and Simmerling, C. (2015) ff14SB: Improving the accuracy of protein side chain and backbone parameters from ff99SB. J. Chem. Theory Comput., 11, 3696-3713.
11. Horn, H.W., Swope, W.C., Pitera, J.W., Madura, J.D., Dick, T.J., Hura, G.L. and Head-Gordon, T. (2004) Development of an improved four-site water model for biomolecular simulations: TIP4P-Ew. J. Chem. Phys., 120, 9665-9678.
12. Dupradeau, F.-Y., Pigache, A., Zaffran, T., Savineau, C., Lelong, R., Grivel, N., Lelong, D., Rosanski, W. and Cieplak, P. (2010) The R.E.D. Tools: Advances in RESP and ESP charge derivation and force field library building. Phys. Chem. Chem. Phys., 12, 7821-7839.
13. Wang, J., Wolf, R.M., Caldwell, J.W., Kollman, P.A. and Case, D.A. (2004) Development and testing of a general amber force field. J. Comput. Chem., 25, 1157-1174.

14. Davidchack, R.L., Handel, R. and Tretyakov, M. (2009) Langevin thermostat for rigid body dynamics. J. Chem. Phys., 130, 234101.
15. Barth, E., Kuczera, K., Leimkuhler, B. and Skeel, R.D. (1995) Algorithms for constrained molecular dynamics. J. Comput. Chem., 16, 1192-1209.
16. Berendsen, H.J.C., Postma, J.P.M., van Gunsteren, W.F., DiNola, A. and Haak, J.R. (1984) Molecular dynamics with coupling to an external bath. J. Chem. Phys., 81, 3684-3690.
17. Case, D.A., Aktulga, H.M., Belfon, K., Ben-Shalom, I.Y., Berryman, J.T., Brozell, S.R., Cerutti, D.S., Cheatham, T.E., Cisneros, I.G.A., Cruzeiro, V.W.D., Darden, T.A., Forouzesh, N., Giambaşu, G., Giese, T., Gilson, M.K., Gohlke, H., Goetz, A.W., Harris, J., Izadi, S., Izmailov, S.A., Kasavajhala, K., Kaymak, M.C., King, E., Kovalenko, A., Kurtzman, T., Lee, T.S., Li, P., Lin, C., Liu, J., Luchko, T., Luo, R., Machado, M., Man, V., Manathunga, M., Merz, K.M., Miao, Y., Mikhailovskii, O., Monard, G., Nguyen, H., O'Hearn, K.A., Onufriev, A., Pan, F., Pantano, S., Qi, R., Rahnamoun, A., Roe, D.R., Roitberg, A., Sagui, C., Schott-Verdugo, S., Shajan, A., Shen, J., Simmerling, C.L., Skrynnikov, N.R., Smith, J., Swails, J., Walker, R.C., Wang, J., Wang, J., Wei, H., Wu, X., Wu, Y., Xiong, Y., Xue, Y., York, D.M., Zhao, S., Zhu, Q. and Kollman, P.A. (2023) Amber.
